# Genome-wide analysis of DUF221 domain-containing gene family in *Oryza* species and identification of its salinity stress-responsive members in rice

**DOI:** 10.1371/journal.pone.0182469

**Published:** 2017-08-28

**Authors:** Showkat Ahmad Ganie, Dipti Ranjan Pani, Tapan Kumar Mondal

**Affiliations:** 1 Division of Genomic Resources, ICAR-National Bureau of Plant Genetic Resources, Pusa Campus, New Delhi, India; 2 NBPGR Base Centre, ICAR-National Rice Research Institute Campus, Cuttack, Orissa, India; Louisiana State University, UNITED STATES

## Abstract

DUF221 domain-containing genes (*DDP* genes) play important roles in developmental biology, hormone signalling transduction, and responses to abiotic stress. Therefore to understand their structural and evolutionary relationship, we did a genome-wide analysis of this important gene family in rice. Further, through comparative genomics, *DDP* genes from *Oryza sativa* subsp. (*indica*), nine different wild species of rice and *Arabidopsis* were also identified. We also found an expansion of the *DDP* gene families in rice and *Arabidopsis* which is due to the segmental duplication events in some of the gene family members. In general, a highly purifying selection was found acting on all the deduced paralogous and orthologous *DDP* gene pairs. The data from microarray and subsequent qRT-PCR analysis revealed that although several *OsDDPs* were differentially regulated under salinity stress, yet *OsDDP6* was upregulated at all the developmental stages in salt tolerant rice genotype, FL478. Interestingly, *OsDDP6* was found to be involved in proline metabolism pathway as indicated by protein network analysis. The diverse gene structures, varied transmembrane topologies and the differential expression patterns implied the functional diversity in *DDP* genes. Therefore, the comprehensive evolutionary analysis of *DDP* genes from different *Oryza* species and *Arabidopsis* performed in this study will provide the basis for further functional validation studies vis-à-vis *DDP* genes of rice and other plant species.

## Introduction

Excess soil salinity is an important abiotic stress that adversely affects growth and development of plants. Rice is one of the most important cereals of the world. In general, rice plant is regarded as sensitive to salinity stress. High salinity stress causes severe impact on millions of hectares of agricultural land worldwide [1, 2]. Accumulation of salts in the irrigated soil is one of the prime factors that lowers water availability to root cells of rice plants due to their reduced osmotic potential, hence diminish growth, development and yield of rice [3,4]. Like other plants, the maintenance of osmotic and ionic homeostasis under salinity stress is a key challenge in rice too, which ultimately defines the salinity tolerance level of a particular rice genotype [5]. Therefore, there is an earnest need to develop salinity stress tolerant rice genotypes to overcome problems of salinity-related yield loss [6,7]. Plants manifest salinity stress responses in several domains such as morphological, physiological, biochemical and ultra-structural changes of cell to various molecular events [8–13]. Hence, the study of expression profiles, physio-chemical and structural properties of specific gene families as well as their expressions under a particular stress condition is the pre-requisite to uncover their specific functional roles [14]. Taking the advantage of sequenced genome, several gene families of rice under different abiotic stresses have been studied. Few examples are sodium/calcium exchanger [15], F-Box [16], SAP (Stress Associated Protein) [17], NAC (NAM, ATAF1/2 and CUC2) transcription factor [18], MADS-box (MCM1-agamous–deficient serum response factor) [19], GF14 [20], DREB (dehydration responsive element binding) [21], SnRK2 [22], TIFY [23], calcium-dependent protein kinase [24], WRKY [25], calcineurin B-like [26], MAPK (mitogen activated protein kinase) [27] and plasma-membrane intrinsic protein (PIP) gene families [28]. However, the unravelling of precise functions of many stress-responsive genes from different plants including *Arabidopsis* and rice remain one of the foremost challenges [29], and such gene products have been categorized as proteins containing hypothetical domains of unknown functions (DUFs). These domains are greatly conserved across genomes [30,31] indicating their biological importance. For example, DUF221 domain containing proteins are exclusively present in eukaryotic genomes [32]. DUF221 family (pfam accession: 02714) belongs to anoctamin superfamily (pfam accession: cl21726), which also contains another family, anoctamin/calcium-activated chloride channels (pfam 04547). DUF221 is homologous to domains present in calcium-activated chloride channels anoctamin/TMEM16 [33]. Although anoctamins are calcium-activated, all of them are not ion channels, some members are phospholipid scrambles which translocate the phospholipids in a lipid bilayer [32]. The DUF221 domain containing proteins (DDPs) are found mostly as transmembrane protein families in combination with other functional domains, as implied by the probable role of DUF221 in membrane integration of the host protein [34]. The first indication for the existence of *DDPs* as gene families in plants came from the model plant *Arabidopsis* [35]. This unique domain has been found in many stress-responsive genes of many plants. For example, this domain has been found to be present in a dehydration-responsive gene, *ERD4*, of *Brassica juncea* [34]; drought associated genes (e.g. LOC_Os12g39320) of rice [36]; osmotic shock-responsive influx cation channel gene, *AtCSC1* in *Arabidopsis*, where this domain helps in the permeability to Ca^2+^ ions under hyperosmotic shock [37]. Recently, Yuan et al. [35] have characterized a hyper-osmolality-gated calcium-permeable channel, *OSCA1* (homolog of *AtCSC1*) from *Arabidopsis* which increases intracellular Ca^2+^ levels under hyperosmotic stress. Since this protein and other members from the same family possess DUF221 domain, it indicates that this domain may also play a direct or indirect roles in osmosensing. This signature domain has also been detected in a vacuolar membrane protein, PenV of *Penicillium chrysogenum* which is associated with beta-lactum biosynthesis [38]. Moreover, this domain has been discovered in the virulence genes of fungus, *Colletotrichum higginsianum* [39]. However, little is known about the functions of DUF221 domain containing genes in plants. Except, Li et al. [40], who have done a preliminary analysis of DUF221 gene family (named as OsOSCA family in their study) in rice, the status of this gene family is largely unexplored in rice too. Therefore, having seen an obvious role of genes possessing DUF221 domain in especially hyperosmotic stress, we did an in-depth study of DUF211 gene family in different *Oryza* species. Additionally, we also studied its transcriptional regulation in relation to salinity stress in rice with the aim of understanding its possible roles in the tolerant glycophytic rice genotype (FL478).

## Materials and methods

### Identification, nomenclature and characterization of *DDP* genes

Arabidopsis DDP amino acid sequences were downloaded from UniPort (http://www.uniprot.org/). Rice DDP amino acid sequences were obtained from RGAP databases (http://rice.plantbiology.msu.edu/). The local Hidden Markov Model-based searches (HMMER: http://hmmer.janelia.org/) was built from all the known DDP proteins. Apart from Hidden Markov Models analysis, we also used ‘DUF221 domain’ as keyword searches in Gramene [41] and Phytozome [42] for the identification of DDP gene family members in the genomes of different plant species. All the retrieved sequences were scanned and curated using Pfam (http://Pfam.sanger.ac.uk/), InterProScan (http://www.ebi.ac.uk/Tools/pfa/iprscan5/) and NCBI Conserved Domain Database (CDD) (http://www.ncbi.nlm.nih.gov/Structure/cdd/wrpsb.cgi) for the authentication of the presence of DUF221 domain.

The number of ESTs (Expression sequence tags) for *OsDDP* genes were determined using data of Rice Genome Annotation Project [43], whereas their functional annotations were verified by AmiGO 2 [44]. To provide a simple nomenclature, members of the studied gene family from rice were named from 1 to10 as per their appearance in chronological order (from top to bottom on the respective chromosomes) on the chromosomes. The genes were denominated as *OsDDP1* to *OsDDP10* (*O**ryza* *s**ativa* DUF221 Domain containing Protein). To denote the splice variants, the Arabic numbers were added after “.” sign. Similar nomenclature was adopted for *DDP* gene family of all other plant species studied herein (**S1 Table**).

Several parameters such as length, isoelectric point (pI) and post-translational modifications and grand average of hydropathicity (GRAVY) values [45] of all the DDP protein sequences of rice, other *Oryza* species (*O*. *barthi*, *O*. *brachyantha*, *O*. *glaberrima*, *O*. *glumaepatula*, *O*. *longistaminata*, *O*. *meridionalis*, *O*. *nivara*, *O*. *punctate*, *O*. *rufipogon* and *O*. *sativa* (*indica*) and *Arabidopsis* were determined using Expasy server (www.expasy.org).

### Chromosomal organization, segmental duplication, detection of introns and exons, alternative splicing

Genomic distribution of *OsDDPs* and *AtDDPs* was depicted using MapChart 2.30 with default parameters [46] and the orientations of these genes were identified from Gramene [41] and TAIR 10 [47] respectively. Segmental duplications of *DDP* gene family of rice and *Arabidopsis* for the detection of homologous genomic regions were determined from their corresponding duplication data in Plant Genome Duplication Database [48]. Alternative splicing of each gene was determined from plant ensemble server.

Number of introns and exons in the individual *DDP* gene were determined by aligning the corresponding genomic and coding sequences. Number of introns in alternative splice forms was determined from the Gramene web server [41]. Synteny blocks were visualized using VISTA (VISualization Tool for Alignments) [49].

### Identification of transmembrane topology and domain organization of DDP proteins from rice and *Arabidopsis*

The putative transmembrane domains and signal peptides for the longest ORF (open reading frame) of each of the DDP proteins were identified by PROTTER version 1.0 [50]. Besides, DUF221 domain and other conserved domains were detected by Pfam (http://Pfam.sanger.ac.uk/), InterProScan (http://www.ebi.ac.uk/Tools/pfa/iprscan5/) and NCBI Conserved Domain Database (CDD) (http://www.ncbi.nlm.nih.gov/Structure/cdd/wrpsb.cgi).

### Motif analysis and phylogenetic relation among DDP proteins

All the OsDDP protein sequences were scanned using MEME Suite 4.10.1/MAST software [51] for the identification of conserved motifs, with the same parameters as used by Mondal et al. [52]. Functional annotations of these motifs were performed using HHpred (http://toolkit.tuebingen.mpg.de/hhpred), and their sequence logos were generated with WebLogo [53]. The phylogenetic relationship among the various DDP proteins was analysed using ClustalW [54] and the dendrogram was constructed using MEGA 6 [55] by neighbour-joining method [56], with all default parameters except a bootstrap of 1000 replicates. The orthologous genes are enlisted in **S2 Table**.

### Estimation of *Ka/Ks* ratio

Segmentally duplicated genes from rice and *Arabidopsis* were used to estimate the extent of selection pressure on them using codeml program based PAL2NAL (http://www.bork.embl.de/pal2nal/). The *Ka/Ks* ratios of orthologous genes of three *OsDDPs* from different plant species as well as orthologues of all *OsDDPs* from the wild species of rice were determined. Likewise, *Ka/Ks* ratios of the orthologues of three *AtDDPs* were estimated from different plant species. Three genes each of rice and *Arabidopsis* were selected on the basis of the presence of their orthologues in all the studied plant species. For each orthologous gene pairs, *Ka/Ks* ratio was estimated, the average ratio values were plotted. The age of the duplicated genes was calculated as done by Jami et al. [57].

### Gene expression analysis with microarray data

Microarray-based expression profiles of *OsDDPs* and *AtDDPs* were retrieved from publicly available Affymetrix microarray data (51 K and 22 K Affymetrix gene chips respectively, experimental Ids: Os-00001 for rice and AT-00120 for *Arabidopsis*). Expression patterns were studied in 29 and 37 different tissues as well as at 9 and 10 developmental stages of rice and *Arabidopsis* respectively, by retrieving the log_2_-transformed affymetrix data on respective arrays using Genevestigator database tool [58]. Likewise, the expression patterns of *DDPs* in salinity tolerant rice cultivar FL478 as well as *Arabidopsis* under salinity stress were studied in roots and shoots by retrieving the corresponding log_2_-transformed fold change values. Heat maps were generated by means of the MeV software package [59] with average linkage hierarchical clustering using Euclidean distance metric for tissues and developmental stages, and Pearson correlation for stress. The expression datasets retrieved from the Genevestigator and subsequently used for generating the heat maps are provided in **S3 Table** and **S4 Table** for rice and *Arabidopsis*, respectively.

### Identification of miRNAs targeting DDPs of rice and its wild species

To predict miRNAs that may target *DDPs*, the individual cDNA sequences of *DDP* genes of rice were used as input in psRNATarget [60] against all the rice mature miRNAs that were reported in miRbase [61]. The predicted miRNAs are enlisted in **S5 Table.**

### Plant material, stress treatment and detection of transcriptional regulation by qRT-PCR (quantitative real time polymerase chain reaction)

The FL478, a salinity tolerant rice cultivar [3], was used for the gene expression study through qRT-PCR. Seeds were surface sterilized with sodium hypochlorite (3%) and were germinated on the germination papers. Then plants were raised up to the reproductive stage under greenhouse conditions in pots. The salt stress (EC of ~10 ds/m) was applied to the pot-grown rice plants for 24 h at different developmental stages namely: tillering, booting, flag leaf, panicle initiation and milk stages (young grain with white liquid). Simultaneously, control samples were obtained at each developmental stage of the rice plant treated with distilled water for 24 h. The tissues after harvesting from the stressed as well as control rice plants were immediately frozen and kept at -80°C until further use.

RNA isolation, cDNA synthesis and qRT-PCR analysis were performed according to the protocol described by Ganie et al. [3]. For qRT-PCR study, 3 biological replicates per sample were used with 3 technical replicates for each biological replicate.

It is worth mentioning here that we used the diluted cDNA (1:100), reverse transcribed from total RNA with oligodT primer using Superscript II (Invitrogen), as a template for quantifying the *OsDDPs*. For the quantification of expression of miRNAs targeting *OsDDPs*, cDNA synthesised from total RNA (as used for *OsDDPs*) instead of cDNA synthesised from miRNA was used. The primers were designed manually with actin as endogenous control. Primers used for qRT-PCR study are given in **S6 Table**. Statistical analyses were conducted using the SAS 9.4 software (SAS Institute Inc., NC, U.S.A).

### *Cis*-element analysis

Almost 2 kb of upstream sequence from the translation start site of all the 10 *OsDPPs* and *DDPs* from different wild rice species were analyzed by PLACE database [62] for abiotic stress-responsive *cis-*elements. Further to determine the interactions with the other proteins, the corresponding protein sequence of differentially regulated OsDDP proteins were analysed using STRING software version 9.1 [63] to reveal their functional interactions with other proteins.

## Results

### Identification and structural analysis of *DDP* gene family in rice, its wild species and *Arabidopsis*

Based on the presence of DUF221 domain, a genome-wide investigation led to identification of 10 members of *DDP* gene family each in *O*. *sativa* (*japonica*), *O*. *barthi*, *O*. *brachyantha*, *O*. *glaberrima*, *O*. *longistaminata*, *O*. *meridionalis*, *O*. *nivara*, *O*. *rufipogon* and *O*. *sativa* (*indica*); whereas only nine members were found in *O*. *glumaepatula* and *O*. *punctata*. All the DDP proteins in rice were found to possess a single DUF221 domain which was also reported by Li et al. [40]. In addition, majority of the identified proteins showed DUF4463 and RSN1_TM domains upstream of DUF221 domain. It was also found that rice *DDPs* displayed similar organization of DUF221 and other two domains when compared with their orthologues in different wild rice species as well as *Arabidopsis*. Further, all the DDP proteins except *OsDDP8* in rice showed positive GRAVY scores indicating their transmembrane nature (**S1 Table)**. Functional annotation further confirmed that DDP proteins were membrane bound proteins.

### Chromosomal distribution and duplication of rice and *Arabidopsis DDP* genes

The distribution of *DDP* genes across rice and *Arabidopsis* genomes was relatively uneven in the sense that not all the chromosomes contained the loci coding for DDPs (**S1A and S1B Fig)**. For example, chromosome 2, 4, 6, 8 and 9 of rice as well as 2 and 5 of *Arabidopsis* did not have any DDP genes. Ten and 15 loci in rice and *Arabidopsis*, identified in our study, encoded for 18 OsDDP and 28 AtDDP proteins respectively, indicating that these genes have undergone alternative splicing. For example, six *DDP* genes in rice and seven *DDP* genes in *Arabidopsis* had alternative splicing (**S1 Table)**. The *DDP* genes from wild species of rice, except *O*. *brachyantha*, *O*. *glaberrima*, *O*. *longistaminata*, *O*. *punctata* and *O*. *sativa* (*indica*), also had alternative splicing as indicated by the disproportion in the number of loci and the protein products (**S1 Table**). The number of *DDP* genes was found to be almost identical among rice and its wild species. Compared to rice, *Arabidopsis* was found to contain 15 *DDP* genes, which could be attributed to the higher number of gene duplication events in latter. In contrast to the number (11) as reported by Li et al. [40], the number of *DDP* genes in rice in our study was found to be only 10. This is because we rejected LOC_Os03g04450 (*OsOSCA4*.*1*) of Li et al. [40] as it failed to show any DUF221 domain by NCBI Conserved Domain Database. Further only one pair of rice *OsDDPs* (20%) was found to have block duplication, while as, no gene pairs were found to be segmentally duplicated (**S1A Fig)**. The block duplicates of *OsDDP3* and *OsDDP10* were found on chromosome 3 and 12 respectively. In case of *Arabidopsis*, six pairs of genes formed by nine genes (60%) were found to be duplicated as block, however like rice, no segmental duplicates could be found. The 10 block duplicates localized on chromosome 1, 3 and 4 with maximum number (4) of duplicated block on chromosome 1 and 4 (**S1B Fig)**. In addition, it was found that almost all the *DDP* gene members were located in the syntenic regions across the different *Oryza* species (**S2 Fig**).

### Phylogenetic analysis among the paralogous and orthologous DDPs

**Fig 1:**
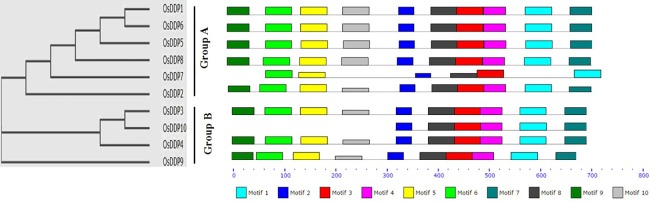
Phylogenetic tree of OsDDP proteins along with their different motifs. Ten different motifs were found in MEME analysis. The proteins, except OsDDP7 in Group A and OsDDP10 in Group B, with the similar motif organization are found in the same groups of tree. Segmental duplicated pair (OsDDP3-OsDDP10) can be seen in the same sub-group. The details of the 10 motifs can be seen in S10 Table.

**Fig 2:**
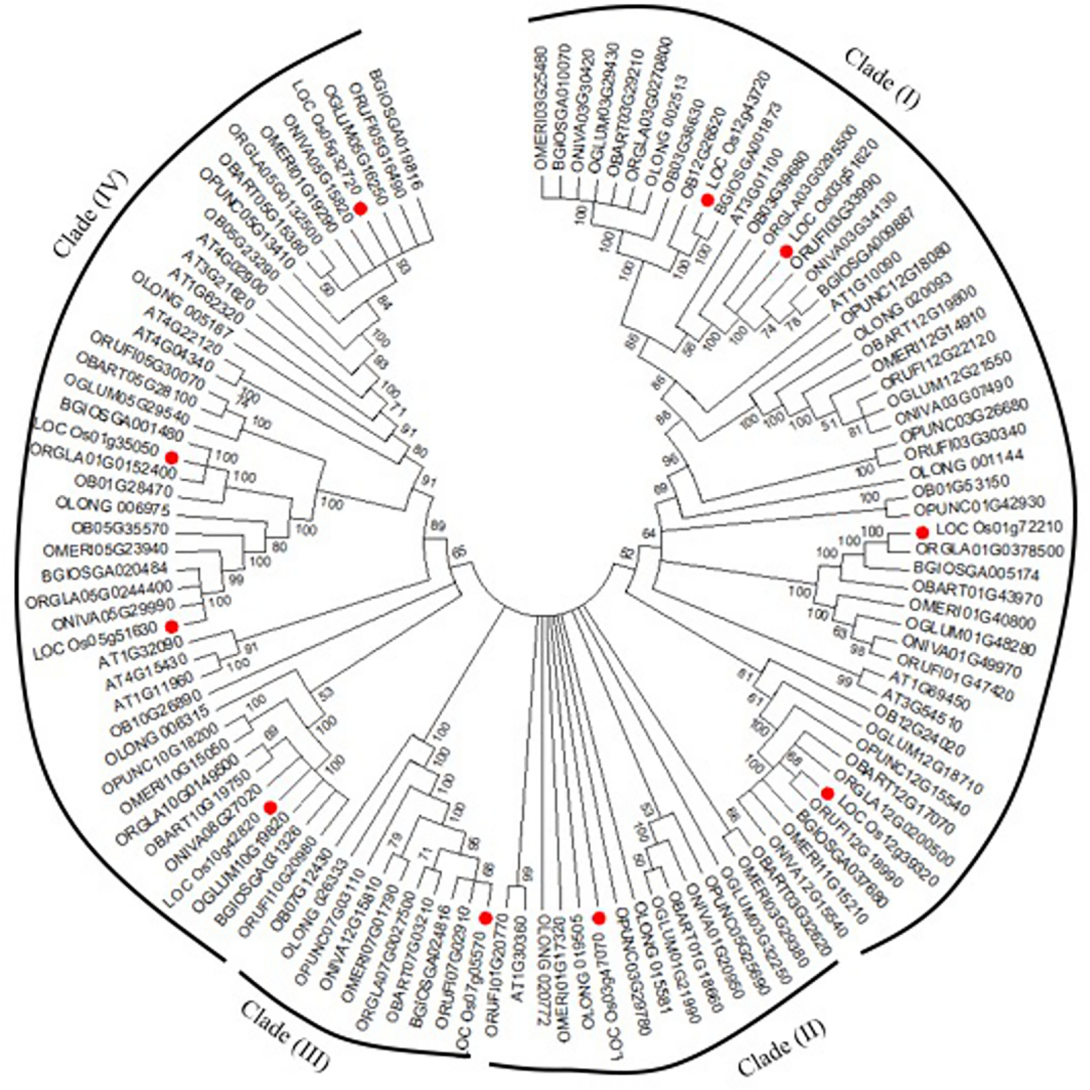
Dendrogram of DDP proteins from rice, different wild rice species, *O*. *sativa* (*indica*) and Arabidopsis. DDP protein sequences were aligned using ClustalW and the dendrogram was generated using MEGA6 software by the Neighbor-joining method with 1000 bootstrap replicates. DDP proteins are categorized into 4 different clades on the basis of sequence homology as described in the text.

With the purpose of evaluating the evolutionary relationship among the DDP proteins of rice, its different wild species and *Arabidopsis*, a phylogenetic analysis was performed. Firstly, a phylogenetic analysis was performed among the 10 rice DDPs (**Fig 1)**. The pair-wise amino acid similarities among OsDDPs varied from 27 to 81% (**S7 Table)**. It can be seen from the **Fig1** that OsDDP1 and OsDDP6 proteins, having the maximum similarity (81%) between them, occurred as the closet pair in Group A of phylogenetic tree, whereas OsDDP1 and OsDDP8 were the farthest in the group due to low similarity (32%). It was also observed that the segmentally duplicated pair, OsDDP3-OsDDP10 occupied the same sub-group. OsDDP proteins with the similar membrane topology or the similar pattern of sequence motifs were found to be present in the same group of the phylogenetic tree. Next, the phylogenetic relationship among the DDP proteins of rice, its wild species and *Arabidopsis* was evaluated. For this, multiple sequence alignment of all DDP proteins was performed to construct a dendrogram that segregated them into four major clades **(Fig 2)**. Based on the similar membrane topology and intron-exon structure, the DDPs from the orthologous species were found to occupy the same clades of dendrogram. DDPs of all the species were distributed in all the 4 clades except *Arabidopsis* and *O*. *glumaepatula* which were not present in the clade III, the smallest among the 4 clades. The clade I was found to contain maximum number of 51 DDP members while as, clade III was represented by the minimum of 10 DDPs. No clade or sub-clade was found to be specific to any particular species. It was observed that OsDDPs from *O*. *sativa* (*japonica*) were more closely related to those of *O*. *sativa* (*indica*), *O*. *glaberrima*, *O*. *rufipogon* and *O*. *barthi*, whereas *O*. *longistaminata* and *O*. *punctata* were found to be less closely related to *O*. *sativa* (*japonica*) sp.

### Estimating the age and selection pressure for duplicated gene pairs

In order to estimate the approximate evolutionary age of segmentally duplicated paralogous of *DDP* gene pairs in *Arabidopsis* and rice, we employed the number of synonymous substitutions per synonymous site (*Ks*). The nucleotide sequences of the duplicated gene pair, *OsDDP3*-*OsDDP10*, in rice have a *Ks* value of 0.8116, indicating that this gene pair might have duplicated~62.4 MYA (million years ago). Likewise, the duplicated gene pairs *AtDDP2*-*AtDDP14*, *AtDDP6*-*AtDDP14*, *AtDDP9*-*AtDDP13*, *AtDDP12*-*AtDDP6* and *AtDDP8*-*AtDDP7* in *Arabidopsis* showed *Ks* values of 1.9492, 3.0995, 0.8802, 2.1362 and 3.1159 respectively, signifying that these block duplications might have occurred approximately 65, 103, 29, 71 and 104 MYA.

**Fig 3:**
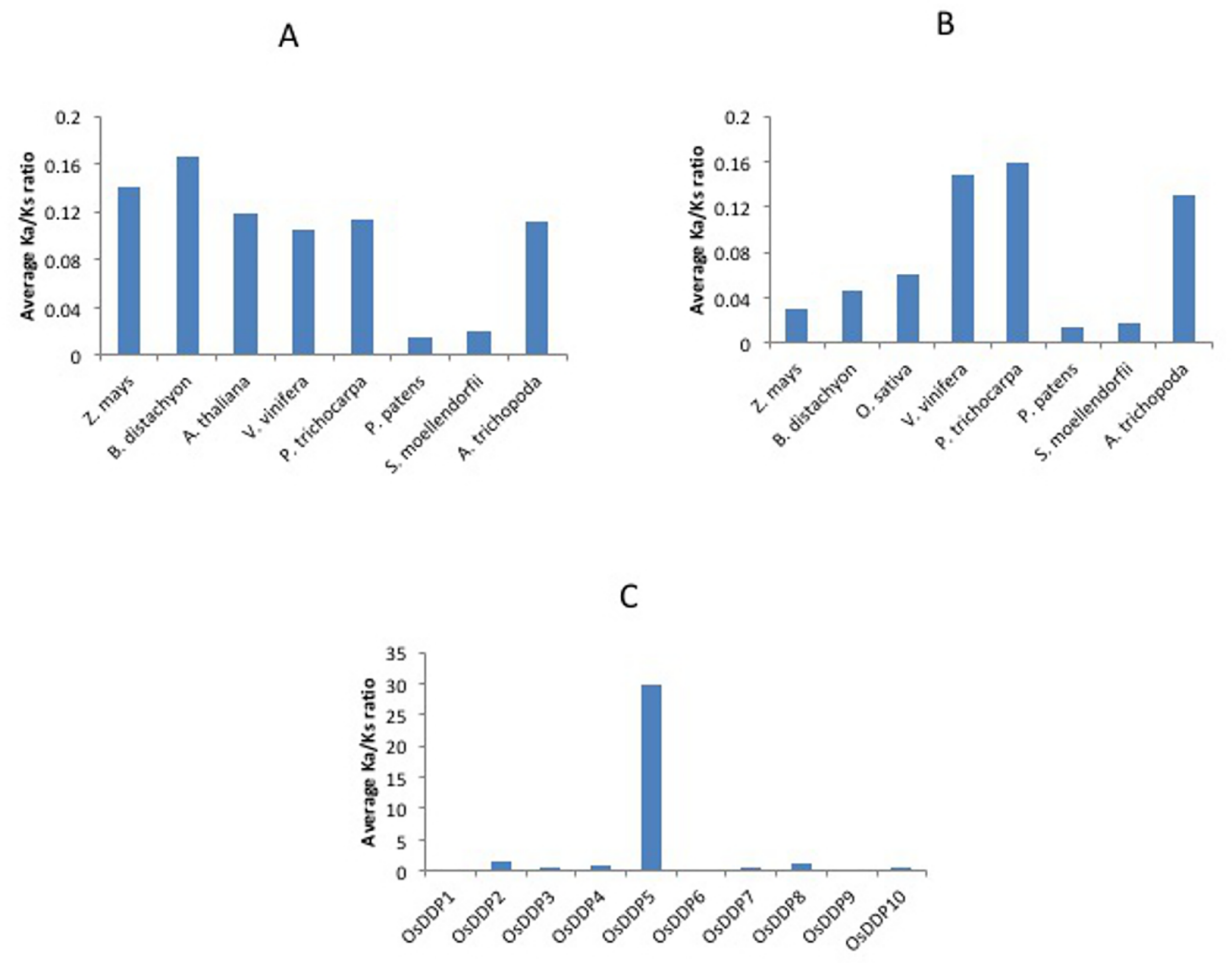
Analysis of evolutionary pressure on *DDP* genes. Averaged *Ka/Ks* ratio between orthologous *DDP* genes of rice (**A**) Arabidopsis (**B**) and different selected plant species. Averaged *Ka/Ks* ratio between orthologous *DDP* genes of rice, its different wild species and *O*. *sativa* (*indica*) rice **(C)**. The *Ka/Ks* ratio of each pair is given in S8 Table.

When *Ka/Ks* for a gene pair is equal to 1, it is said to be going through neutral evolution, while gene pairs undergoing positive or negative purifying selection have *Ka/Ks* >1 or <1, respectively [64]. All the rice and *Arabidopsis* segmentally duplicated paralogous *DDP* gene pairs as well as their orthologous gene pairs in different plant species exhibited *Ka/Ks* <1 (**Fig 3** and **S8 Table)**. However, five *OsDDPs* (*OsDDP2*, *OsDDP4*, *OsDDP5*, *OsDDP7* and *OsDDP8*) exhibited a *Ka/Ks >*1 with some of their wild rice orthologues, whereas the rest five *OsDDPs* (*OsDDP1*, *OsDDP3*, *OsDDP6*, *OsDDP9* and *OsDDP10*) showed *Ka/Ks <*1 for all their corresponding wild rice orthologues (**S8 Table)**.

### Sequence conservation among the DDP proteins

DUF221 domain is significantly conserved across the different eukaryotic genomes [30]. To find out its conservation, DDP protein sequences from rice were aligned. It was found that DUF221 domain region was the highly conserved region across the OsDDP sequences (**S3 Fig**). Apart from DUF221 domain, many other localized regions were also found to be conserved in DDPs across the different species (**S4 Fig)**. To confirm the identity of these locally conserved regions, MEME search helped us to identify 10 different motifs which are fairly conserved among rice DDP protein sequences (**Fig 1, S9** and **S10 Tables)**. To affirm if the motifs obtained from the MEME analysis are similar to any of the known protein motifs, we did HHpred analysis. It was observed that these novel motifs did not show any significant similarity with the known motifs (**S10 Table)**.

### *Cis-*motifs in the *DDP* gene promoters

An extensive analysis of 2kb upstream promoter region (from the translation initiation codon) of *DDP* genes from rice, its nine different wild species and *O*. *sativa* (*indica*) facilitated the identification of conserved and over-represented consensus *cis*-motifs involved in abiotic stresses especially salt and osmotic/dehydration stresses. Twenty different salt and osmotic stress-responsive *cis-*elements were clearly found in the promoters of *DDPs* (**S11 Table**). Among them, MYCCONSENSUSAT is the most enriched *cis-*element in all the studied promoter sequences, followed by ACGTATERD1 and MYBCORE. These three *cis*-elements along with other three elements (GT1GMSCAM4, MYB1AT and MYB2CONSENSUSAT) were present in the promoter regions of all the *DDP* sequences. Interestingly, *cis*-elements ABREATRD22 was found to be absent from the promoters of all *O*. *nivara* and *O*. *longistaminata* DPP genes. None of the promoters of *DDP* gene possessed all the 20 identified motifs. The average number of the identified *cis*-elements was found to be almost similar in all the *DDPs* of rice and its wild species (**S5 Fig**).

### miRNAs-*DDP* gene family networking

In an attempt to find out the miRNAs targeting the *DDP* family members of rice and its wild species, psRNATarget predicted only a few members of *DDP* gene family from all the *Oryza* species that were targeted by conserved miRNAs (**S5 Table**). For an example, in rice, *OsDDP6* and *OsDDP10* were targeted by miRNAs belonging to three different families. Five members of osa-miR818 family (osa-miR818a, b, c, d and e) as well as osa-miR1436 were predicted to target *OsDDP6* by cleavage and inhibition of translation respectively, whereas osa-miR6248 was predicted to inhibit the translation of *OsDDP10*. Similarly, in case of wild rice species, the number of *DDP* family members targeted by miRNAs ranged from minimum of two each in *O*. *barthi* and *O*. *glumaepatula* to maximum of five in *O*. *meridionalis*, whereas the number of miRNAs targeting the different *DDP* gene family members ranged from minimum of three (targeting four different *DDP* genes in *O*. *longistaminata*) to maximum of ten (targeting five different *DDP* genes in *O*. *meridionalis*). Interestingly, miR1436 and different members of miR818 family were found to target the *DDP* genes from most of the *Oryza* species studied except *O*. *longistaminata*, *O*. *punctata* and *O*. *brachyantha*. Further, it was found that a particular *DDP* gene (e.g. *OsDDP6*) was targeted by multiple miRNAs.

### Transmembrane topology and intron-exon structure of *DDPs* from rice, wild species of rice and *Arabidopsis*

*DDP* genes encode integral membrane proteins with multiple predicted transmembrane (TM) helices to act as transport channels. Topological structure prediction using Protter software and hydrophobicity analyses showed the presence of TM helices in all DDPs (**S6 Fig)**. The number of TM helices varied from a minimum of 7 (OsDDP2) to maximum of 11 (OsDDP10) in rice, and from 9 (AtDDP1, AtDDP2, AtDDP4, AtDDP5, AtDDP6, AtDDP7, AtDDP9, AtDDP11, AtDDP12, AtDDP13 and AtDDP15) to 11 (AtDDP3, AtDDP8 and AtDDP10) in *Arabidopsis*. Similarly for wild species of rice, it was varied from 7 (OloDDP5, OloDDP7) to 11 (ObaDDP5, OglaDDP2, OglDDP5, OmeDDP2, OsiDDP1, OsiDDP2, OsiDDP5). TM helices in DDPs were found to be located in the amino acid sequence between 6–27, 87–111, 145–165, 357–381, 401–429, 449–468, 549–580, 601–621 and 627–647. Besides the TM helices, another modular architecture of DDPs was the ‘loop region’ which was found to be located between the amino acid residues of 166 to 356 amino acid residues. It was observed from the TM topology that all the DDPs had two distinct clusters of TM helices joined by a large hydrophilic intracellular loop. However, as an exception, the two clusters of TM helices in ObarDDP1 protein of *O*. *barthi* were joined by an extracellular loop. In general, lesser number of TM helices was located in the N-terminal cluster than in the C-terminal cluster. DUF221 domain represents the C-terminal cluster containing in general six predicted TM helices.

Structural analysis of all *DDP* genes implied that the number of introns varied from minimum of 4 in rice (*OsDDP3*) to maximum of 16 in *Arabidopsis* (*AtDDP5*) (**S7 Fig)**. In *Arabidopsis*, *AtDDP15* was the only member found to be intron-less. The average number of introns in *DDP* gene family were found to be conserved (approximately nine introns) among all the studied species with a maximum average of 11.2 introns in *O*. *meridionalis* and a minimum of nine introns in case of *O*. *glaberrima*. Additionally, variations in lengths of exons, introns and UTRs (untranslated region) were observed. None of the *DDP* genes of rice were found to lack either 5´ or 3´ UTRs, whereas in *Arabidopsis AtDDP5* and *AtDDP7* were found to be lacking 5´ UTR, and another pair (*AtDDP6* and *AtDDP11*) lacking both 5´ and 3´ UTRs. However, the absence of either one or both the UTRs was found more frequently among the 10 species of *Oryza* with *O*. *glaberrima*, *O*. *longistaminata* and *O*. *sativa* subsp. *indica* lacking both the UTRs. The segmentally duplicated *DDP* genes of both rice and *Arabidopsis* were found to have a different organisation as well as dissimilar number and length of introns and exons. Orthologous genes with almost similar number of introns and exons were found to be present in the same clade of dendrogram. However, the organisation of introns and exons was not coherent in all of them.

### Expression profiles of *OsDDPs* and *AtDDPs*

**Fig 4:**
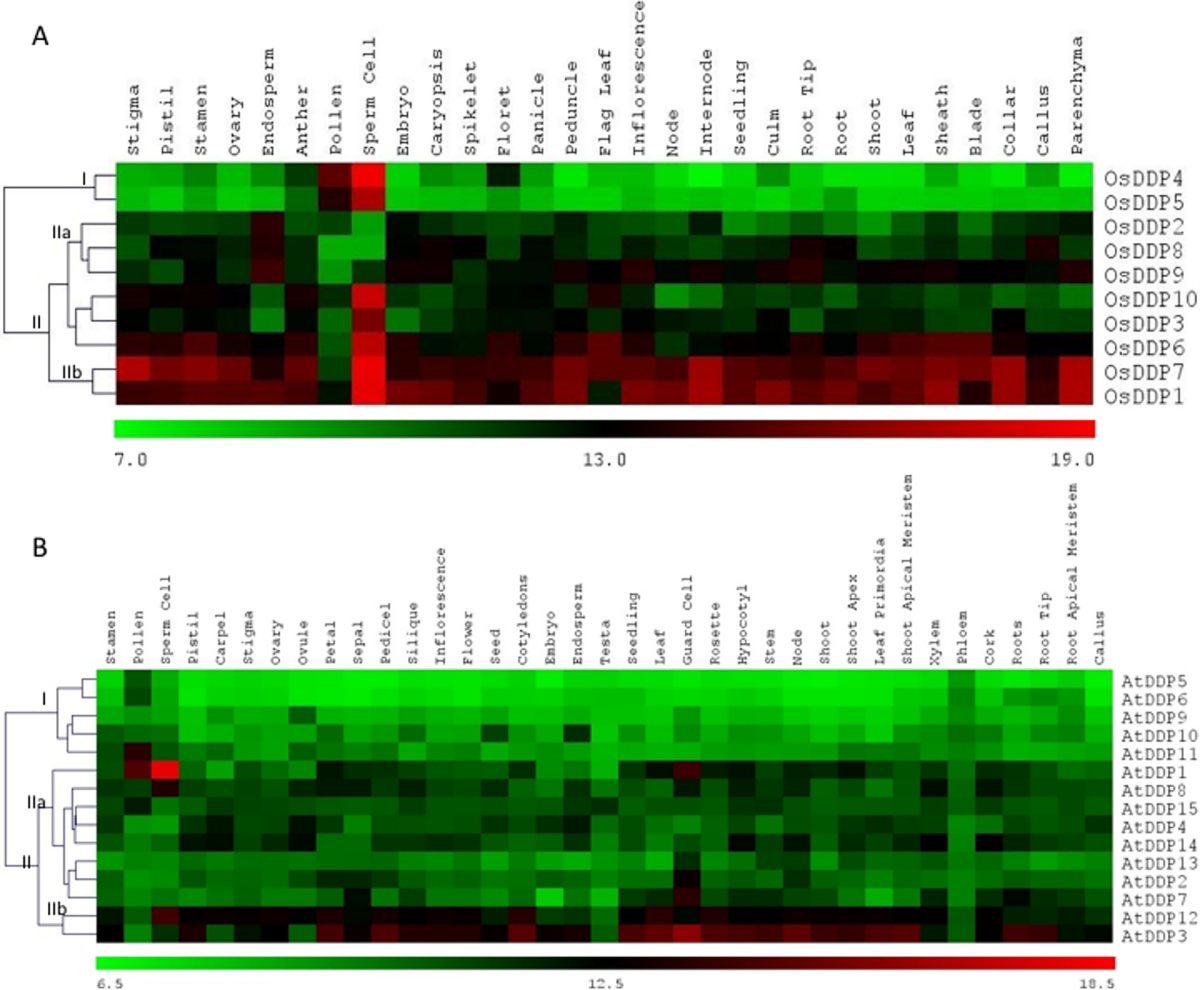
Microarray-based expression pattern of *DDP* genes in different tissues/organs. Expression profile of *DDP* genes generated from microarray data indifferent tissues/organs of **(A)** rice and **(B)** Arabidopsis. The heatmaps represent hierarchical clustering (using Euclidean distance metric) of average log signal values of *DDP* genes and were generated using MeV software package. The color bar below the heat maps represent relative expression values with green color representing the lowest expression while as, red the highest expression level. Cluster-grouping represented by Roman numbers followed by letters as discussed in the text.

Microarray-based spatio-temporal expression profiles of *OsDDPs* and *AtDDPs* in various tissues and organs, at different developmental stages as well as under salinity stress were analysed using publicly available microarray data of Genevestigator. In both rice as well as *Arabidopsis*, the microarray expression profiling of *DDP* gene family showed that the members of this family had varied expression patterns. For an example, in rice, *OsDDP4* and *OsDDP5* (in cluster I) exhibited the lowest level of expression in all the studied tissues (except sperm cells, in which all the *OsDDPs* showed high expression except *OsDDP2* and *OsDDP8*) (**Fig 4A**). However, in contrast, the expression of *OsDDP1* and *OsDDP7* (in cluster-IIb) was the highest in all the tissues. The expression of all other genes in cluster-IIa was almost low among all the tissue. Some of the *OsDDPs* were found to exhibit a moderately high to high expression level specific to certain tissues. They were *OsDDP3*, *OsDDP10*, *OsDDP5* and *OsDDP4* (also showed moderately high expression in pollen tissue) which showed high sperm cell-specific expression; *OsDDP2*, *OsDDP8* (specific to callus and root tip) and *OsDDP9* showed moderately high expression specific to endosperm and some vegetative tissues.

In *Arabidopsis*, however, the expression of *DDPs* in different tissues was not as diverse as was observed in rice. In general, a very low level of expression was observed for all the *AtDDPs* among all the tissues except *AtDDP3* and *AtDDP12* (in cluster-IIb of **Fig 4B**) which showed moderately high level of expression in most of the tissues. The expression of *AtDDPs* in cluster-I was found to be uniformly lowest. It was also found that while most of the *AtDDPs* exhibited a near uniformly low level of expression, higher tissue-specific expression of some *AtDDPs* was also observed. For instance, *AtDDP1* was found to be specifically expressed in guard cells and in some male reproductive tissues such as pollen and sperm cell. Similarly, the expression of *AtDDP7* was also found to be guard cell specific.

**Fig 5:**
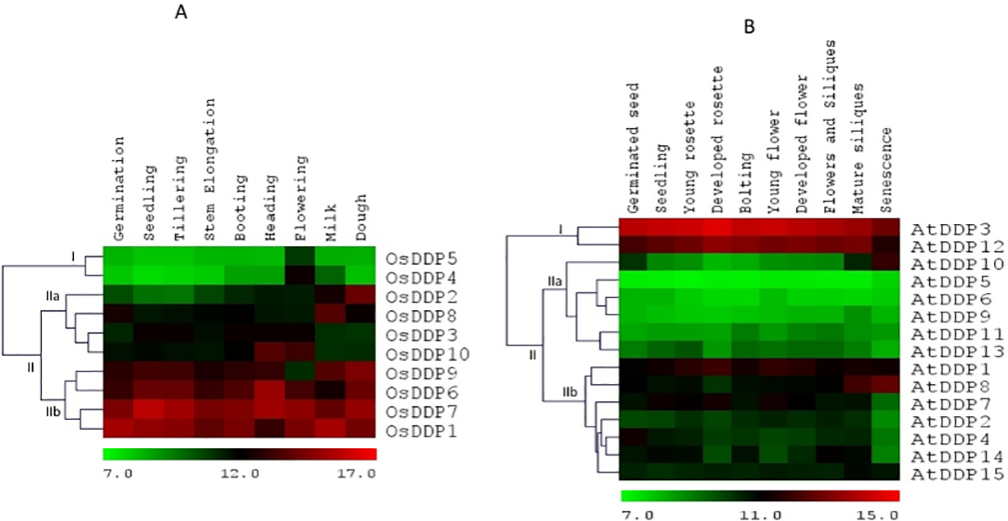
Microarray-based expression pattern of *DDP* genes during development. Heat maps showing expression patterns of *DDP* genes from rice (**A)** and Arabidopsis (**B**) at different developmental stages as indicated. Heat maps represent hierarchical clustering (using Euclidean distance metric) of average log signal values of *DDP* genes and were generated using MeV software package. The color bar below the heat maps represent relative expression values with green color representing the lowest expression while as, red the highest expression level. Cluster-grouping represented by Roman numbers followed by letters as discussed in the text.

Besides, the microarray-based expression of rice and *Arabidopsis DDPs* were also studied at different stages of development. Like their expression in different rice tissues, *OsDDP4* and *OsDDP5* (in cluster-I of **Fig 5A**) exhibited the lowest level of expression across the different stages of development too. Likewise, the expression of *OsDDP1*, *OsDDP7* and *OsDDP6* were also the highest and near constant across the 9 developmental stages. These 3 genes were highly expressed *OsDDP9* in cluster-IIb. All the other genes in cluster IIa showed almost uniform moderately low level of expression.

In *Arabidopsis*, the expression of *AtDDP3* and *AtDDP12* (in cluster-Ia of **Fig 5B**) was the uniformly higher across the 10 different developmental stages than other genes. On the other hand, except the moderately high expression of *AtDDP10* only at senescence stage, all the genes in cluster-IIa showed very low level of expression across the different developmental conditions with *AtDDP5* showed the lowest level of expression. Expression of *AtDDP1*, *AtDDP7* and *AtDDP8* (in cluster-IIb) was found to be specific only to some developmental stages. All other genes in this cluster showed moderately low level of expression.

**Fig 6:**
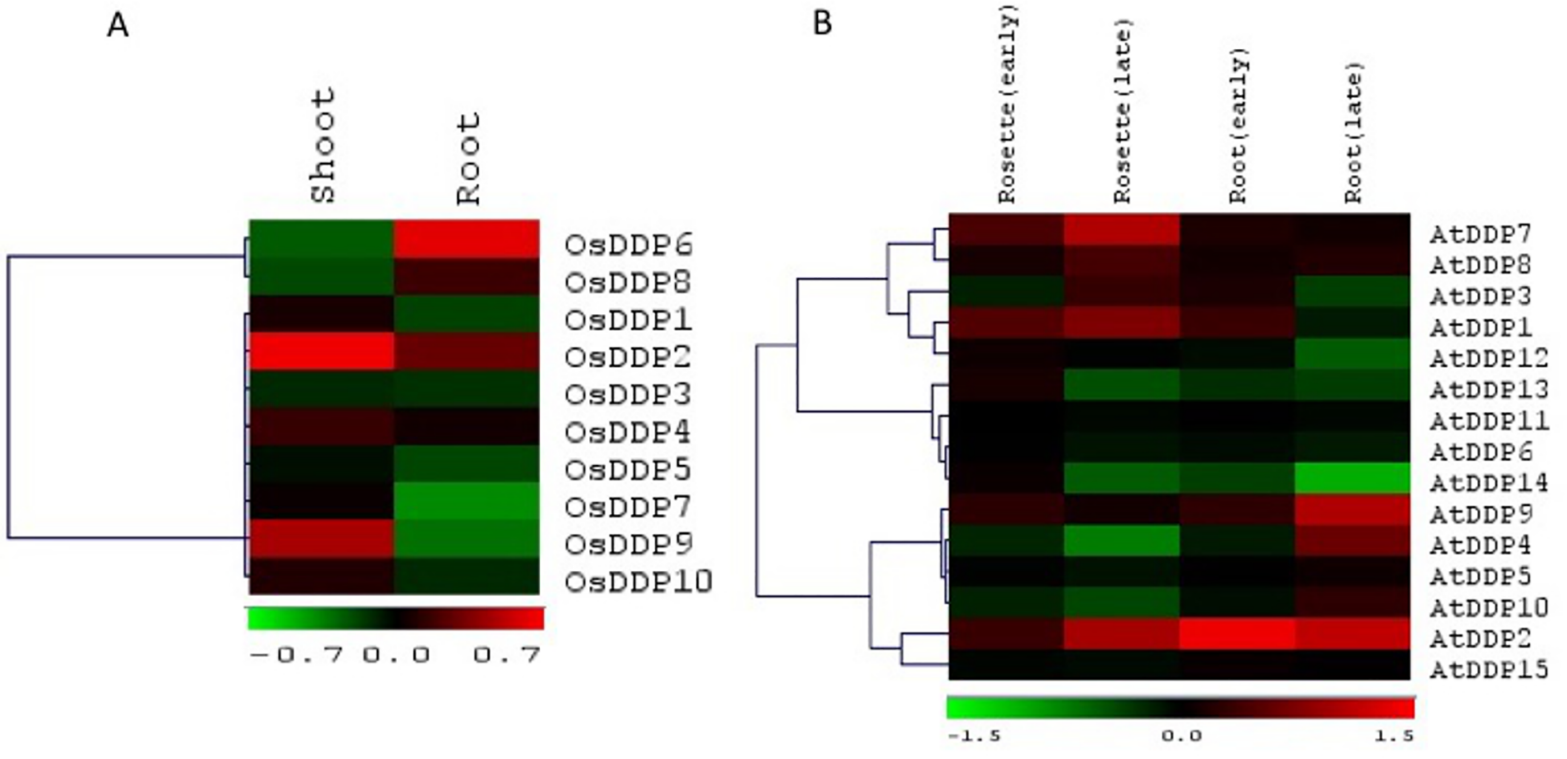
Microarray-based expression pattern of *DDP* genes under salinity stress. Expression analyses of *DDP* genes of rice (**A)** and Arabidopsis **(B)** under salt stress in shoots and roots as indicated above the heat map. Weighted average linkage method and Pearson correlation distance metric were used for hierarchical clustering of *DDP* genes. The color bar below the heat maps represent relative expression values with green color representing the lowest expression while as, red the highest expression level.

In order to identify the members that are responsive to salinity stress, we analysed the expression of *OsDDPs* and *AtDDPs* under salinity stress. In case of rice, we exploited the microarray data for the roots and shoots of salt tolerant cultivar FL478 under salt stress. As shown in **Fig 6A,** *OsDDP2* and *OsDDP9* were highly up-regulated in shoots, while as, their expressions in roots were moderately up-regulated and down-regulated respectively. However, *OsDDP4* was up-regulated and down-regulated in shoots and roots respectively. In the roots of FL478, *OsDDP6* was highly up-regulated, whereas its expression in shoots was very low. Similarly, the expression of *OsDDP8* was moderately up-regulated in roots but down-regulated in shoots. All other *OsDDPs* displayed low to moderately low level of expression both in roots and shoots under salinity stress. In general, all *OsDDPs* except *OsDDP6* and *OsDDP8* showed higher level of expression in shoots than roots of FL478.

In *Arabidopsis*, microarray-based expression data of *AtDDPs* in root and shoot (rosette) was retrieved from AtGenExpress, Genevestigator. As shown in **Fig 6B**, the expression of *AtDDP1*, *AtDDP2*, *AtDDP7* and *AtDDP8* in rosette leaf of *Arabidopsis* was up-regulated from the early phase to late phase of salt stress, whereas the expression of the rest *AtDDPs* was down-regulated, except *AtDDP7*which exhibited unaltered expression in roots. In the roots, however, the expression of *AtDDP4*, *AtDDP9* and *AtDDP10* was seen to be up regulated from the early to late periods of the stress, whereas *AtDDP1¸ AtDDP2*, *AtDDP12* and *AtDDP14* were found to be down-regulated from the early to late phase of the stress. Salt stress was not however found to alter the expression of *AtDDP12* in rosette. All other *AtDDPs* were found to be expressed at a low level under salinity in the roots. Moreover, the expression of *AtDDP2*, under salinity, was found to be the highest, whereas the expression of *AtDDP14* was the lowest, among all the *AtDDPs* in both rosette and shoot. Additionally, the expression of four genes such as *AtDDP5*, *AtDDP6*, *AtDDP11* and *AtDDP15* was found to be significantly invariable in both rosette and roots under salinity stress.

### qRT-PCR-based expression analysis of *OsDDPs* and of miRNAs targeting *OsDDPs*

**Fig 7:**
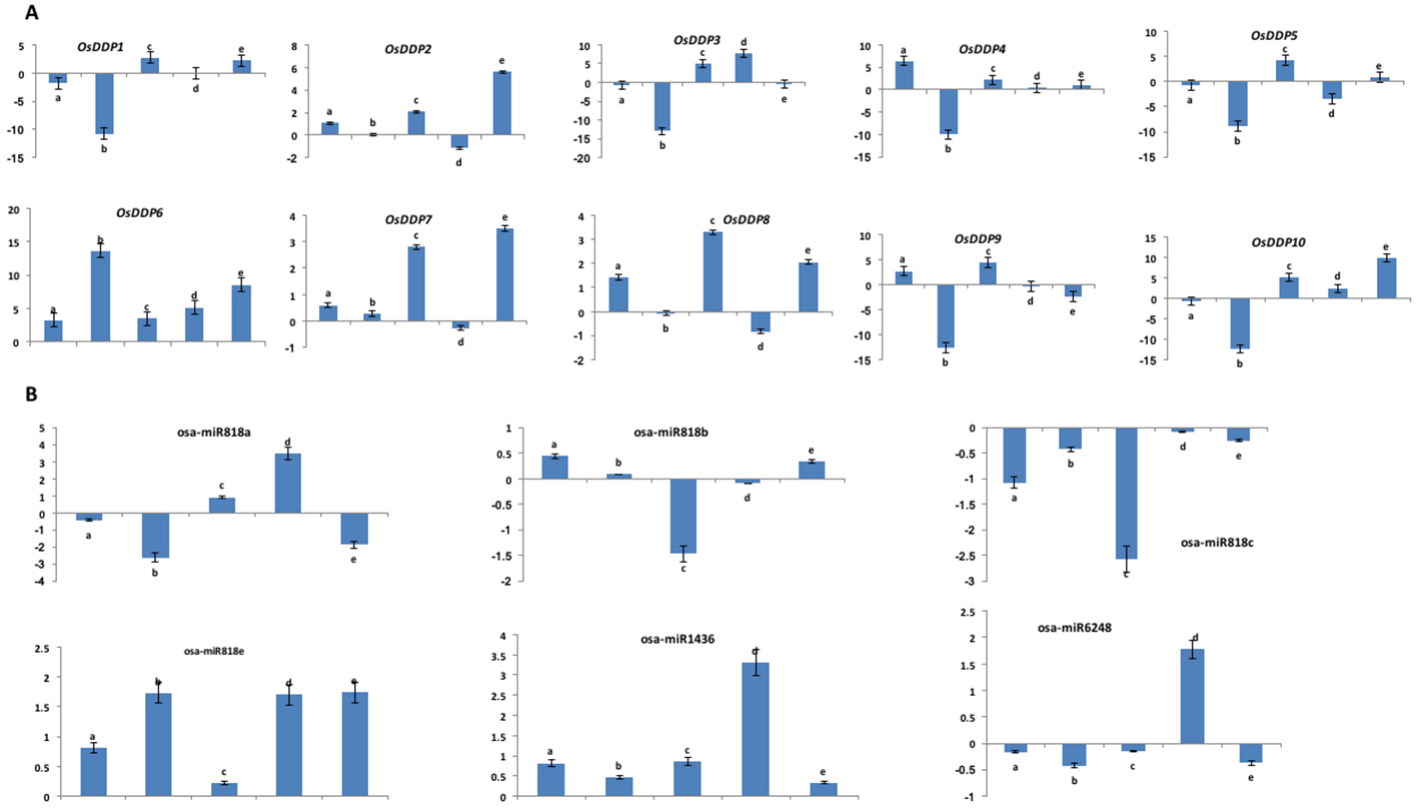
qRT-PCR based expression profiles. Mean fold change (log_2_ scale) in expression of all *DDP* gene members **(A)** and miRNAs predicted for *DDP* genes **(B)** in salt tolerant FL478 rice genotype at different developmental stages under salinity stress. Values on Y-axis represent the relative transcript abundance (log_2_ scale). qRT-PCR analysis of both *DDP* genes and miRNAs was done with cDNA template generated from the harvested tissues of salt stressed (200 mM NaCl) and unstressed (control) plants as mentioned in materials and methods. As osa-miR818d could not be amplified, it was not hence included in the qRT-PCR analysis. Only osa-miR818c showed the opposite expression as that of its target (*OsDDP6*). Error bars represent the standard deviation (±), n = 3. Different letters indicate significant differences at *P ≤ 0*.*05*. Different alphabets indicate different developmental stages (a = tillering, b = booting, c = flag leaf, d = panicle initiation, and e = grain with white milk stages).

To gain insights into the better understanding about how *OsDDP* genes changed their expression in response to salinity stress as well as to validate their altered gene expression as shown by microarray-based expression patterns in FL478, we analyzed the expression profiles of the 10 *OsDDPs* as well as six miRNAs by qRT-PCR at different developmental stages of FL478. Our results indicated that all the 10 genes were up-regulated at one or more developmental stages of FL478 under stress conditions (**Fig 7A)**. Among them, *OsDDP6* was found to be up-regulated across all the developmental stages. On the contrary, microarray data indicated *OsDDP2* to be the most responsive to salinity. All the genes, except *OsDDP3* and *OsDDP9* were up-regulated at flag leaf and milk developmental stages, whereas all the genes barring *OsDDP6* and *OsDDP7* were down-regulated at the booting stage. At the vegetative phase (tillering), only *OsDDP1*, *OsDDP3*, *OsDDP5* and *OsDDP10* were found to be down-regulated. It was also observed that *OsDDP4* displayed the highest level of expression at tillering stage, *OsDDP6* at booting and milk stages, *OsDDP3* at panicle initiation and flag leaf stages, whereas *OsDDP10* showed the highest level of expression at milk stage. Moreover, as shown in **S1 Table**, EST analysis showed that the number of ESTs for the *OsDDP* gene members varied from 5 (*OsDDP5*) to 73 (*OsDDP8*).

The expression analysis of different miRNAs targeting *DDP* genes showed that none of the studied miRNAs, except osa-miR818c, showed a completely reverse expression pattern to that of their targets across all the developmental stages under salinity stress (**Fig 7B)**. Instead, they showed reverse abundance in their transcript levels only at particular stages of development. For an example, osa-miR818e and osa-miR1436 showed up-regulation across all the developmental stages studied which was not coherent with the expression level of their target gene *OsDDP6*. Among the miRNAs targeting *OsDDP6*, only osa-miR818c showed reverse regulation at all the stages. Similarly, osa-miR6248 showed the reverse level of expression as that of its target *OsDDP10*, but only at flag-leaf and milk stages.

**Fig 8:**
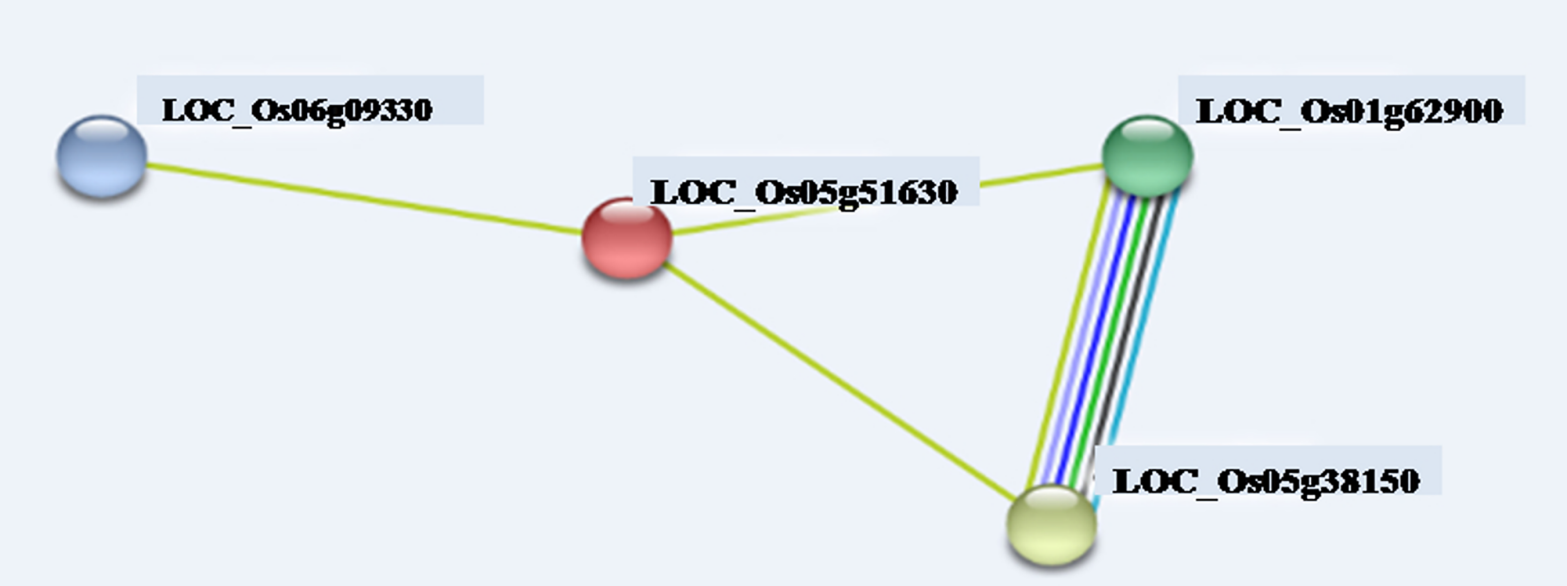
Protein interaction network of LOC_Os05g51630. In the network generated by STRINGV9.1, each node represents a protein and each edge represent an interaction, coloured by evidence type (see STRING website for colour legend). The original graphic output was modified to name the LOC number of the gene.

Having seen that *OsDDP6* was up-regulated at all the developmental stages, we were then interested to know its interaction with the other genes. Therefore, to identify the protein network that consists of proteins interacting with the differentially expressed gene i.e. *OsDDP6* [LOC_Os05g51630], direct and indirect interactions between these proteins were derived using the STRING search tool. The network connecting up-regulated proteins is shown in **Fig 8**, and indicates that there were four nodes (representing proteins) and four edges (representing interactions). The interacted proteins were ubiquitin-conjugating enzyme [LOC_Os06g09330], amino acid kinase [LOC_Os01g62900] and P5CS (plays a key role in proline biosynthesis, leading to osmoregulation in plants). This interaction clearly indicated that *OsDDP6* is involved in salinity tolerance. It was also found that there were two KEGG pathways in the network namely ‘arginine and proline metabolism’ and other one was amino acid metabolism.

### Expression of genes lying on duplicated segments of rice and *Arabidopsis* genomes

The rigorous search in the Plant Genome Duplication Database [48] revealed that 2 and 9 genes were present on duplicated segments of chromosomes in rice and *Arabidopsis* respectively (**S1 Fig**). In order to understand the functional diversification of duplicated *DDP* genes, the microarray-based expression profiles for 1 and 6 pairs of duplicated genes in rice and *Arabidopsis* respectively, were compared under salinity stress, in different tissues as well as across several developmental stages (**S8 Fig**). Based on their microarray based expression profiles, the two segmentally duplicated paralogous genes (*OsDDP3* and *OsDDP10*) in rice showed almost identical expression pattern in all the tissues and across the developmental conditions. However, they exhibited expression divergence under the stress conditions due to the specifically moderate expression of *OsDDP10* in shoots. In *Arabidopsis*, the duplicated gene pairs showed high divergence in expression as compared to rice. The paralogous pairs *AtDDP2-AtDDP14* and *AtDDP9-AtDDP13* showed divergence only with respect to stress. A high divergence in expression domain of *AtDDP12-AtDDP6* gene pair was observed, as the expression of *AtDDP12* was specific to different vegetative and reproductive tissues as well as had a slightly different expression pattern than *AtDDP6* under stress conditions. Comparison of expression patterns of *AtDDP8-AtDDP7* pair revealed that this pair had undergone divergence in expression due to the highly specific expression of only *AtDDP8* at late reproductive and senescence stages. Likewise, *AtDDP13-AtDDP11* also differentially expressed under different vegetative and reproductive tissues. Expression profile of *AtDDP6-AtDDP14* duplicated pair exhibited tissue and early stress specific expression.

## Discussion

Plants acquire the tolerance to environmental stresses by orchestrating the changes of various events including gene expression [65]. However, the exact functions of some gene products, categorized as proteins containing hypothetical domains of unknown functions (DUFs), are not studied well. One such domain is DUF221. The highly conserved *DDPs* are osmoregulatory cation channels permeable to calcium [35, 37], and therefore may be associated with the abiotic stress responses such as salinity.

Since most of the orthologous *DDP* gene family members were located on the same order of chromosome among the different *Oryza* species (**S2 Table**), it was intriguing to know if these orthologous genes were present as syntenic blocks in chromosomal regions. We found through VISTA alignments that almost all the orthologous genes were present in syntenic blocks. For example, in case of *OsDDP3* (**S2 Fig**), almost 1.5 kb intergenic region on 5′-end and 3.5 kb intergenic region on 3′-end were found to be highly conserved across the *Oryza* species, indicating this region to be a syntenic block. However, considerable differences were found for *O*. *punctata* and *O*. *brachyantha* genomes which could be due to their different genome types, BB- and FF-genomes respectively. The similar length of conserved intergenic region surrounding *MIR319a* locus in orthologous species of *Arabidopsis* has already been reported [66]. Further, to assure the genetic colinearity in the analyzed syntenic region, we found that a gene (*Os03g0673600*) next to the 5′-end of the *OsDDP3* was consistently present in the similar order of location across the *Oryza* species with BB- and FF-genomes, confirming our assumption to form the synthenic region. The similarity among these genes found next to the 5′-ends of the *OsDDP3* orthologues ranged from 70–95%.

The higher number with different lengths of *DDP* transcripts per gene loci (**S7 Fig, S1 Table**) identified in different *Oryza* species in this study indicates the presence of splice variants that may be associated with functional diversity of *DDP*s. The duplicated gene pairs of rice and *Arabidopsis* (except *AtDDP7* and *AtDDP8*) were found to have different number of splice forms which may add to the divergence in their expression. The alternatively spliced DDP transcripts could be regulated in tissue-, developmental-, or stress-specific manner and hence allow the organisms to fine-tune the gene expression more efficiently [67].

DUF221 domain has been reported to be highly conserved in sequence across the plant genomes [30], which is also confirmed in our study (**S3 Fig**). The other locally conserved regions (motifs) other than DUF221 domain (**S9 Table**) identified through the multiple sequence alignments of DDPs did not show a considerable similarity with any of the known protein motifs. However, they may act as scaffolds for the DDPs and be associated with the calcium transport by some means, thereby adding to the functional divergence of DDPs.

All DDPs are transmembrane calcium permeable channels. Their transmembrane nature was confirmed by higher GRAVY scores, which is a characteristic physiochemical property of transmembrane proteins [45]. The membrane topologies and motif patterns of OsDDP channel proteins were in agreement with their clustering in the phylogenetic tree (**Fig 1)**. Proteins within a single group (Group A or B) of phylogenetic tree shared the similar pattern of different sequence motifs and membrane topologies except OsDDP7 and OsDDP10 in Group A and B respectively. In general, cytoplasmic loops of Group A differed from that of Group B, except OsDDP2, possess 4 folds as compared to 3 folds (except OsDDP3) in Group B (**S6 Fig**). The clustering of OsDDPs was found to be corroborated with the clustering of OsOSCAs as obtained by Li et al. [40]. Similar type of results for NCX membrane-protein family have also been obtained very recently [15]. As reported by of Hou et al. [37], DUF221 domain was found to represent the C-terminal cluster of TM helices by us too.

To infer the further structural changes in the *DDP* genes during the course of evolution, the intron-exon structures of all *DDP* genes were analysed. Although the average number of introns in *DDP* gene family was found to be conserved among all the studied species, a high variation in structural organisation and size of introns as well as exons was found (**S7 Fig**), implying that these gene structures might have undergone shuffling during the course of evolution [68]. Similar diversification in structural organisation of auxin transporter gene families has been documented in maize recently [69]. Further, the expression divergence of segmental duplicated *DDP* genes of both rice and *Arabidopsis* was found to be complemented by the diversified structural organisation of their introns and exons.

The phylogenetic analysis of DDP orthologues from rice, its wild species and *Arabidopsis* showed that almost all the orthologous DDPs, which shared the similar structural organisations, clustered together with a convincing bootstrap value (1000) (**Fig 2**). The dendrogram also revealed that almost all the segmental duplicates of *Arabidopsis* were clustered together into single clade (clade IV). This outcome further substantiates the gene duplication in *Arabidopsis* during evolution, which might eventually allow the protein functional diversity by adaptive evolution [70]. However, the duplicated pair (OsDDP3-OsDDP10) of rice was found scattered in clade I and clade II, which might possibly be due to the more sequence homology between *OsDDP10* of this pair and its orthologous sequences which all were found to be in the same clade. Based on their related structural organization, several DDP proteins from rice and *Arabidopsis* were found to be in the same subgroups implying a common ancestral origin. Similar results with phylogeny are also documented elsewhere [71, 72]. Moreover, despite the fair degree of homology among the *O*. *sativa* (*japonica*), different wild rice species as well as *O*. *sativa* (*indica*), it was inferred from the dendrogram that *japonica* sub-species was more closely related to *indica* sub-species, *O*. *glaberrima*, *O*. *rufipogon* and *O*. *barthi*. These species were found phylogenetically closer in some other earlier studies [40,73,74]. The phylogenetic results obtained here are in agreement with the fact that even closely related *Oryza* species harbour remarkable changes in the genome [74].

Duplications inplant genomes play a central role in species diversification, and hence generating the platform needed for adaptive evolution [75]. The difference in the number of *DDP* genes in the studied plant species prompted us to address if this difference could be attributed to the gene duplication. The number of *DDP* genes was found to be almost identical in rice (with a single segmental duplication in *DDP* gene family) and its wild species (**S1 Table)**. However, *Arabidopsis* genome, although almost three times smaller than that of rice, was found to contain 5 *DDP* genes more than rice genome, which could be ascribed to the higher number of clad-specific genomic segmental duplication events indicot *Arabidopsis* [76,77]. The similar expansion of different gene families in *Arabidopsis* as compared to rice is well documented in literature [19,71,78]. Similarly, segmental duplications have been reported in various other gene families of rice [15,19,72] and *Arabidopsis* [15,29,79]. It is notable to cite that members of *DDP* gene family in segmental duplicated regions of rice and *Arabidopsis* shared 50–78% identity at the amino acid sequence level (**S7 Table)**. Similar level of similarity has also been observed among the duplicated genes of other gene families [19,57].

The gene pairs present on the segmentally duplicated regions of chromosome can acquire functional diversity in terms of neo-functionalization or pseudo-functionalization [80,81]. To address the diversity in the function of duplicated *DDP* gene pairs of rice and *Arabidopsis*, their expression patterns in different tissues as well as developmental stages and under salinity stress were compared (**S8 Fig**). The expression divergence of duplicated gene pair in rice during stress indicates that the duplication is significant for the stress response. The divergence in expression of duplicated genes in some other gene families of rice has also been reported [19, 72]. In *Arabidopsis*, the exhibition of expression divergence by all the duplicated paralogues indicates that they have undergone neo-functionalization or sub-functionalization. Neo-functionalization in duplicated *Arabidopsis* genes has also been reported [82]. The divergence in expression of segmentally duplicated orthologous *DDP* pairs in rice and *Arabidopsis*is is evident from the fact that all these duplicated gene pairs tend to be derived from the duplication events that date back from the appearance of the crucifers to divergence between monocots and dicots (29–104 MYA). Hence, our data corroborates with the fact that the degree of expression divergence of duplicates is proportional to their divergence times [83].

The approximate age of segmentally duplicated *DDP* paralogues of rice and *Arabidopsis* was estimated from their corresponding *Ks* values [84]. The approximate age (64.2 MY) of duplicated gene pair (*OsDDP3-OsDDP10*) of rice implicates that this duplication dates back to the divergence of poaceae from the common ancestor ~55–70 MYA [85]. The approximate ages of 29, 65 and 71 MY of gene pairs *AtDDP9*-*AtDDP13*, *AtDDP2-AtDDP14* and *AtDDP12-AtDDP6* indicated that duplication of these pairs could have occurred before the appearance of crucifers~24–40 MYA [86] (**S8 Table)**.

From the *Ka/Ks* analysis, it was observed that all the duplicated paralogous gene pairs of rice and *Arabidopsis* undergo purifying (negative) selection, as all of them showed *Ka/Ks* <1 (**S8 Table**). The orthologous gene pairs from both the species were also found to be under purifying selection. However, twenty orthologous *DDP* gene pairs of rice and some of its wild species exhibited a *Ka/Ks* >1, indicating a strong positive selection for these pairs. The reason for this high ratio could not be known, however, it might be due to the changes that cultivated rice had accumulated during the course of domestication and divergence from its wild species [87]. The purifying selection on this gene family indicates its crucial role in the plant biology, and therefore unfits in the form of alterations in this gene family would be eliminated by default [88].

To get the insights into the spatio-temporal expression patterns of the *DDP* genes in rice and its different wild species under abiotic stress conditions, detection of regulatory abiotic stress-responsive *cis*-elements in their promoter regions are pre-requisite. Therefore, we restricted ourselves to find only stress related *cis*-elements in the *DDP* genes of rice and its different orthologous of different *Oryza* species. The presence of 20 salt and osmotic stress-responsive *cis-*elements in the promoters of *DDPs* (**S11 Table**) indicated the indispensable role of this gene family in response to such environmental constraints. The high enrichment of dehydration-responsive *cis*-elements such as, MYCCONSENSUSAT [89], ACGTATERD1 [90] and MYBCORE [91] in all the analyzed *DDPs* supports their role in salt and osmotic stress. The average number of the identified *cis*-elements was found to be almost similar in all the *DDPs* of rice and its wild species which might also indicate at their purifying selection as signified by the low *Ka/Ks* values of their orthologues. Moreover, the promoter regions of segmentally duplicated gene pair (*OsDDP3-OsDDP10*) in rice showed a different pattern (both in terms of type and number of motifs) of the identified *cis*-motifs (**S11 Table),** which might be a key factor in determining their expression divergence. The identified *cis*-elements might alter the expression of *DDP* genes in association with their putative transcription factors (TFs) which could ultimately culminate in the stress tolerance through the modulation of intracellular Ca^2+^ levels [92]. The identified motifs can be further analysed through in-depth molecular studies for understanding the transcriptional regulation of *DDP* genes by the putative TFs that bind such motifs.

Next, we attempted to identify if there was any miRNA-target module among the *DDPs*. Only 2 members (*OsDDP6* and *OsDDP10*) were predicted to be targeted by miRNAs belonging to 3 different families (**S5 Table)**. *OsDDP6* was likely targeted by the members of osa-miR818 family (except osa-miR818f) and by osa-miR1436; whereas osa-miR6248 was predicted to target *OsDDP10*. Members of osa-miR818 are important for the regulating post embryonic development [93]. The miR818 of barley and osa-miR1436, targeting *OsDDP6*, are reported to be possibly regulated by abiotic stress such as drought and salt [94,95]. Likewise, osa-miR6248 has been reported to be differentially regulated under arsenate stress [96]. Hence, these results implicate that *OsDDP6* and *OsDDP10* might play an important role in abiotic stress responses. Additionally, the divergence in expression of *OsDDP10* from its segmentally duplicated partner *OsDDP3* might also be in part due to its regulation by osa-miR1436. Further, we also predicted few more *DDP* ortholouges in wild species of rice that were targeted by miRNAs (**S5 Table)**. Interestingly, most of the *DDP* members from the rice and different wild rice species, targeted by the same miRNA, were found to be orthologues of each other (**S2 Table)**. This indicated that different orthologues had conserved their regulation by same miRNA even during the course of domestication of rice. Targeting of *DDP* genes from the eight out of 11studied *Oryza* species only by miR1436 and different members of miR818 family suggested the functional redundancy of these miRNAs in regulating the Ca^2+^ levels across different species under different developmental and stress conditions. The finding that a particular *DDP* gene was targeted by multiple miRNAs is supported by the fact that a single gene can be regulated by multiple miRNAs [97,98] and therefore suggests that these miRNAs act in a functionally redundant manner [99].

Analyzing the important characteristic of gene expression pattern across the broad spectrum of different tissues, developmental stages and stress conditions would provide a vital insight into the physiological and developmental functions of *DDPs*. Hence, we exploited the microarray datasets for knowing the possible functions of *DDPs* from rice and *Arabidopsis*. It was found that while many *DDPs* maintain their transcripts at either constantly high or low levels across different tissues and developmental stages, several others expressed specifically in different tissues and at particular stages of development. This type of expression pattern has also been obtained for other gene families of rice and *Arabidopsis* [19,79]. In rice, the high level of expression of almost all the *OsDDPs*, especially *OsDDP3*, *OsDDP10*, *OsDDP5* and *OsDDP4*, in sperm cells indicated their role in reproduction and hence in the early embryogenesis (**Fig 4A**). Uniformly, high expression of *OsDDP1*, *OsDDP7* and *OsDDP6* in all studied tissues implicates that these 3 genes might be involved in fundamental physiological functions. The highly specific expression of *OsDDP4* in pollen tissue highlights its role in male reproductive development. The endosperm-specific moderately high expression of *OsDDP2*, *OsDDP8* and *OsDDP9* indicates towards their involvement in the nourishment and the development of embryo. The expression of *DDPs* in different tissues of *Arabidopsis*, however, was not as diverse and as high as observed in rice (**Fig 4B**). The similar difference in the expression pattern of histone chaperone gene families of rice and *Arabidopsis* was also observed [100]. The moderately high expression of *AtDDP3* and *AtDDP12* across almost all the studied tissues specified their commitment to some basal cellular functions. In addition, the guard cell-specific expression of *AtDDP1* and *AtDDP7* pointed out their possible role in stomata dynamics. Besides, the very high level of expression of *AtDDP1* in sperm cell and pollen is an indicative of its concern with the male reproductive development.

The microarray-based expression patterns of *OsDDPs* and *AtDDPs* were also studied at different stages of development (**Fig 5A and 5B)**. In rice, the highly expressed *OsDDP1*, *OsDDP7* and *OsDDP6* during developmental stages corroborated with their constantly high expression across the different tissues, and hence pointing towards their role in the development of rice plant. Moreover, the higher level of expression of *OsDDP2*, *OsDDP8* and *OsDDP10* specifically during the reproductive stages is an indication of their probable role in grain yield. Although the expression of *OsDDP3* was uniform and moderately high across the developmental stages, the specific expression of *OsDDP10* during the heading stage might partly be accredited to the segmental duplication and subsequent divergence in this gene pair. Similarly, in case of *Arabidopsis*, the highest expression of *AtDDP3* and *AtDDP12* across the development of the plant was in agreement with their similar degree of expression across the tissues. The high level of expression of *AtDDP8* and *AtDDP10* at the senescence stage indicates that they can be regulated by ethylene and abscisic acid, the two principal hormones involved in senescence [101, 102]. Owing to their possible roles in senescence, these two genes may also play a role in augmenting the grain yield [103] and in protecting the plants from stress conditions [104]. In addition, the specific expression of *AtDDP1*and *AtDDP7*during the vegetative and early reproductive stages indicated their possible involvement in transition from vegetative to reproductive phase of development.

Analysis of microarray-based expression profiles of *DDP* gene family members in rice and *Arabidopsis* led to the identification of some individual members which might play crucial roles for the tolerance of salinity stress (Fig **6A** and **6B**). In rice, microarray-based expression profiles were analyzed in the root and shoot of FL478 under salinity stress condition. The up-regulation of *OsDDP6* and *OsDDP2* in roots under salinity indicates that the two genes might act in concert as the sensors of overwhelming salinity at the site of contact with the surrounding environment.

We also validated the expression profiles of rice *DDP* genes by qRT-PCR at different developmental stages of FL478 (a salinity stress tolerant genotype) under salinity stress. It was found that the salinity tolerance of FL478 at different developmental stages might be partly contributed by one or more members of *OsDDP* family highly expressing at those stages (**Fig 7A)**. The high level of expression of these calcium channel encoding genes is reminiscent of the fact that cytosolic Ca^2+^ concentration is crucial for regulating the response to stress conditions [92,105,106]. The upregulation of *OsDDP2*, *OsDDP6*, *OsDDP4*, *OsDDP9* and *OsDDP10* under different stages of development in this study corroborated with their microarray-based expression. However, in general *OsDDP6* was found to be exhibiting the higher level of expression under salinity stress than *OsDDP2* (showing the high overall expression in both root and shoot in microarray-based data) which might be due to the fine-tuning in the regulation of its expression by osa-miR1436 as well as different members of osa-miR818. Hence, *OsDDP6* can prove as an important candidate in salt tolerance breeding of susceptible rice genotypes. Like their microarray-based expression pattern, the expression of segmentally duplicated gene pair such as *OsDDP3*-*OsDDP10* was also found to be diverged across different developmental stages. Further, the upregulation of most of the *OsDDPs* at the flag leaf (particularly *OsDDP3*) and milk stage (especially *OsDDP6* and *OsDDP10*) indicates their crucial role in regulating the grain filling and grain size, and hence in the yield under salinity stress. Moreover, the up-regulation of only *OsDDP6* and *OsDDP7* at booting stage indicates that these 2 members partly take care of tolerating the salt stress at this stage. The highest expression of *OsDDP3* at the panicle initiation/emergence indicates its role at this relatively more salinity sensitive stage, and hence might contribute to the more grain numbers under salinity. The contributory duty of salinity tolerance at vegetative stage has been bestowed especially to *OsDDP4*.

The expression of *OsDDP2*, *OsDDP6*, *OsDDP7*, *OsDDP9* and *OsDDP10* at vegetative stage was found to be supported by the results of Li et al. [40], who studied the expression of *OsDDPs* (as *OsOSCAs* in their study) only at four-leaf stage of rice plants (the early tillering stage) under abiotic stress conditions. Though all the mentioned genes were up-regulated under salinity stress in their study too, the level of expression and the down-regulation of *OsDDP1* in our study might be due to the different rice genotypes used. Further, the results from EST analysis of *OsDDPs* (**S1 Table**) were almost in accordance with our expression profiling results, as the gene members which had low number of ESTs mostly exhibited low level of expression than those with higher number of ESTs. However, some discrepancies in this relationship might be ascribed to the different rice genotype in our study. In spite of the differential regulation of the *DPP* genes under salinity stress found in this study, more comprehensive molecular and biochemical characterization of putative *DDP* gene members needs to be done so as to establish their role in salinity and other stress conditions.

Moreover, the expression profiles of all miRNAs, baring osa-miR818c, showed the similar trends as shown by their targets *OsDDP6* and *OsDDP10* under salinity stress conditions (**Fig 7B)**. Although osa-miR818e and osa-miR1436 were predicted to target *OsDDP6* (up-regulated under salinity at all the developmental stages), they were found probably not to be regulating its transcript levels as indicated by their up-regulation during salinity. In contrast, the down-regulation of osa-miR818c across all the developmental stages under salinity stress indicated that *OsDDP6* might be the target of osa-miR818c. The down-regulation of osa-miR818a and osa-miR818b at different developmental conditions may possibly augment the role of osa-miR818c in negatively regulating the transcript levels of *OsDDP6*. Such negative regulation was also found in case of osa-miR393a and its target gene *TIR1* under salinity stress (3). In addition, the down-regulation of osa-miR6248 at flag-leaf and milk stages implied that this miRNA might be regulating its target *OsDDP10* at only these two specific stages, whereas its regulation at other developmental stages might be attributed to some other aspects such as promoter methylation [3]. The miRNAs found herein that showed altered expression than their target genes under salinity stress, however, must be analysed by functional genomics studies for verifying the regulation of *OsDDP6* and *OsDDP10* by them.

It is noteworthy to mention here that we used cDNA reverse transcribed from total RNA with oligo dT primer (using SuperScript II) as a template for quantifying both the *OsDDPs* and the respective miRNAs. The quantification of miRNAs using cDNA prepared from total RNA is reported for the first time which was possible as the studied miRNAs were located either in exons or UTRs, which gave us a chance to exploit the usefulness of this technique. This is quite legitimate because miRNA transcripts possess a polyA tail [107, 108] that can be primed with oligo dT in SuperScript II. Additionally, miRNA genes are also transcribed by RNA Pol II like any other protein coding genes [109]. Therefore, this approach of quantifying miRNA transcripts may prove economical and more efficient than what is normally done using costly commercial kits (using mature miRNA sequence as one primer and a universal primer as another). The efficiency of this approach can be understood by that any probe (most commonly mature miRNA), for example in RNA blot, for quantifying the transcript levels of a particular member of a family (that has identical mature sequences in all or most of its members) would or would not give the information specifically about the miRNA member that was actually aimed at. Therefore, to avoid such bottlenecks, the allele specific primers can be designed from the distinct and specific regions of corresponding member after alignment of all the members (precursor sequences) of a miRNA family that have identical mature sequences. Mature sequence can be used as one primer (forward/reverse) and the other primer of the pair can be designed from a region specifically. However, it is also must to mention that this approach has a drawback of not quantifying the miRNAs that are intronic.

In conclusion, this study presents an in-depth account of the *DDP* gene family in rice as well its comprehensive comparative evaluation in different wild species of rice and *Arabidopsis*. The comparative genomics and phylogenetic analysis revealed the fair degree of conservation of this family in the form of high purifying selection. This study also encourages further in-depth molecular investigations targeting some of the differentially regulated *OsDDP* members (*OsDDP2* and *OsDDP6*) and the miRNAs (osa-miR818c) for enhancing the performance of rice and other plant species under high salinity leading to the subsequent agricultural development.

## Supporting information

S1 Fig Genomic distribution of *DDP* genes. (**A)** Chromosomal distribution and segmental duplication of rice *DDP* genes. (**B)** Genomic distribution and expansion of *DDP* genes on Arabidopsis chromosomes. Bars on chromosomes denote the genes while as, red spots on the chromosomes are centromeres. Gene segmental duplications are shown with dotted lines between the duplicated gene pairs.
(TIF)

S2 Fig VISTA comparison of *DPP3* gene among the 10 different species of *Oryza*. Conserved regions are indicated in pink (90% identity over a 100-bp window). The relative position of the Os-DDP3 and synteny block region is also indicated.
(TIFF)

S3 Fig Sequence conservation of DUF221 domain in OsDDP proteins. Multiple sequence alignment of DUF221 domain from DDP proteins of rice. The bar line indicates the DUF221 domain. The gene ids can be seen in S2 Table.
(TIF)

S4 Fig Sequence conservation in DDP proteins. Multiple sequence alignment of **DDP proteins** of selected species is generated using CLUSTAL X and visualized in Jalview. The gene ids of the different species can be seen in S2 Table.
(TIF)

S5 Fig *cis-*element abundance in *DDP* genes of rice and its wild species. Bar diagram showing that the average number of *cis*-elements in the 2 kb promoter regions of *DDP* genes of rice and its wild species as well as *O*. *sativa* (*indica*) is almost uniform.
(TIF)

S6 Fig Predicted topological structures of DDP proteins from rice, wild species of rice and Arabidopsis. Predicted topological structures of all DDP family members of rice, wild rice and Arabidopsis. Topological structures were predicted using Protter v1.0. For splice variants, topological architecture for only the largest transcript has been provided. Finger-like projections represent loops joining two clusters of TMs. The right side of each structure represents the extra-cellular region while as, left side the intra-cellular region.
(JPG)

S7 Fig Intron-exon structure DDP proteins from rice, wild species of rice and Arabidopsis. Typical structure of different *DDP* genes. Lengths are drawn according to the size of genes. Black boxes indicate the coding exons, lines joining them indicate the introns which were not drawn to scale. The white color boxes represent the non-coding exons, whereas the white boxes at the ends represent the UTRs. It can be seen that the average number of introns in DDP gene families of different species was found to be conserved (approximately 9 introns). However, frequent length changes in exons, introns and UTRs can be seen.
(TIFF)

S8 Fig Expression pattern of duplicated *DDP* genes. The line graphs represent the microarray-based expression profiles for one and six pairs of duplicated genes in rice **(A)** and Arabidopsis (**B**) respectively, under salinity, in different tissues as well as across several developmental stages. The expression profile of every duplicated gene pair is shown in three different line graphs to represent tissues, developmental stages and salinity from left to right.
(TIFF)

S1 Table Details of putative *DDP* genes of rice, wild species of rice and *Arabidopsis*. (DOCX)

S2 Table List of *DDP* genes of rice, Arabidopsis and their ortholouges in different plant species with their genomic location and percent identity. (XLSX)

S3 Table Microarray-based expression values retrieved from Genevestig at or for *OsDDP* across different tissues, developmental stages and under salinity stress condition. (XLSX)

S4 Table Microarray-based expression values retrieved from Genevestig at or for *DDP g*enes of Arabidopsis across different tissues, developmental stages and under salinity stress condition. (XLSX)

S5 Table List of miRNAs predicted by psRNA target for *DDP* genes of different *Oryza* species. (XLSX)

S6 Table Details of different primer sequences used in the qRT-PCR study of *DDPs* and the miRNA genes. (XLSX)

S7 Table Percent similarity matrix showing the percent identity for rice and *Arabidopsis* DDP proteins. (XLSX)

S8 Table The *Ka/Ks* ratios for orthologous *DDP* genes of rice (in different plant species including wild species of rice) and *Arabidopsis* (in different plant species). The *Ka/Ks* ratios for segmental duplicates of rice and *Arabidopsis* are also given.
(XLSX)

S9 Table Putative motifs identified from OsDDP proteins using MEME. The sequence logos were generated using WebLogo.
(DOCX)

S10 Table Similarity of motifs identified by MEME analysis in 10 OsDDPs with the known protein domains as analysed by HHPred analysis. (DOCX)

S11 Table List of different putative *cis*-elements found in the 2 kb promoter regions of *DDP* gene family of different rice species. (XLSX)
