# Supplementary figures and images for: Genome-wide analysis of DUF221 domain-containing gene family in Oryza species and identification of its salinity stress-responsive members in rice
Source: PLoS One. 2017 Aug 28;12(8):e0182469. doi: 10.1371/journal.pone.0182469 (PMC5573286; doi:10.1371/journal.pone.0182469)

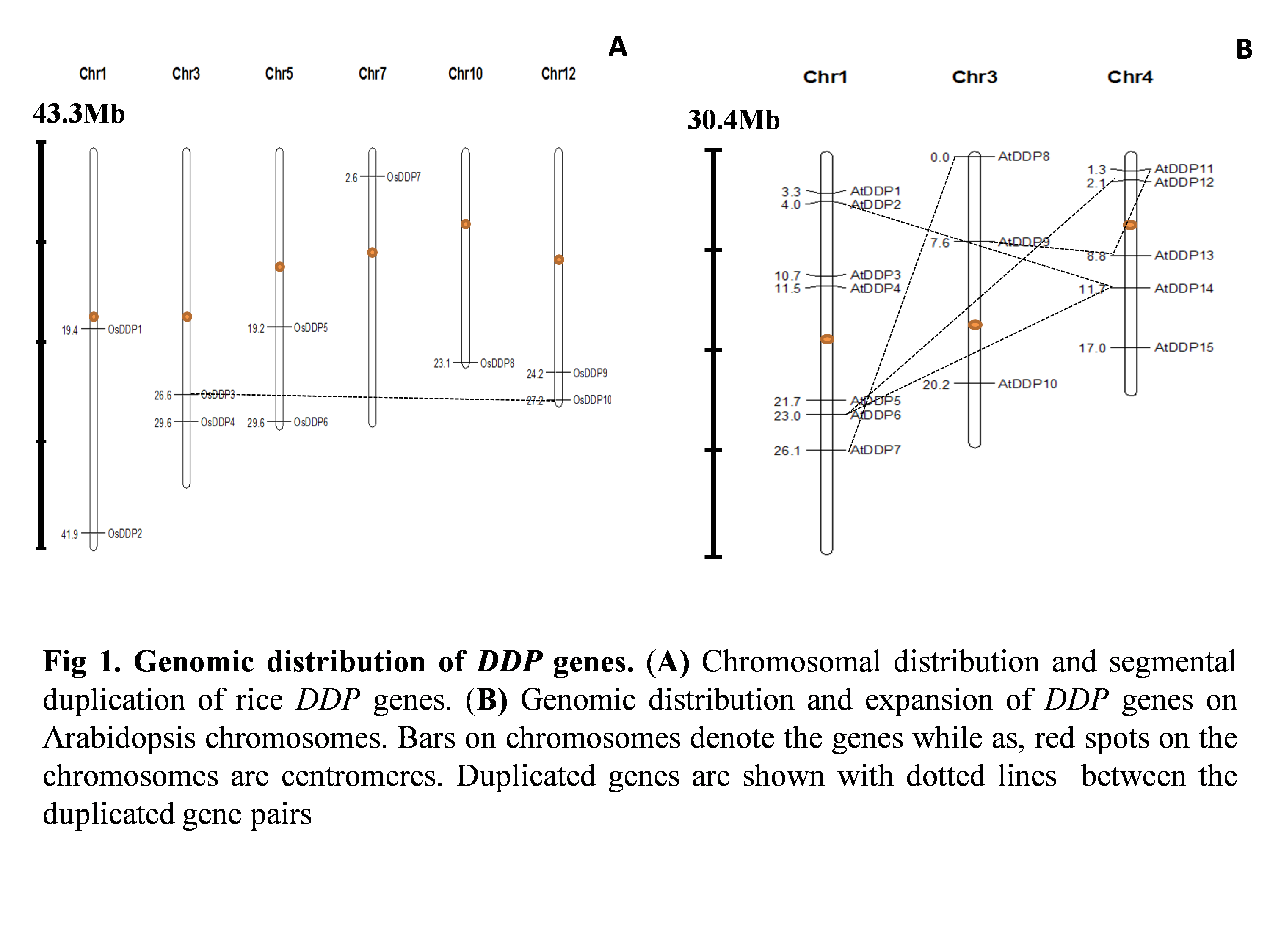

Supplement: S1 Fig — (A) Chromosomal distribution and segmental duplication of rice DDP genes. (B) Genomic distribution and expansion of DDP genes on Arabidopsis chromosomes. Bars on chromosomes denote the genes while as, red spots on the chromosomes are centromeres. Gene segmental duplications are shown with dotted lines between the duplicated gene pairs. (TIF) [file pone.0182469.s001.tif]

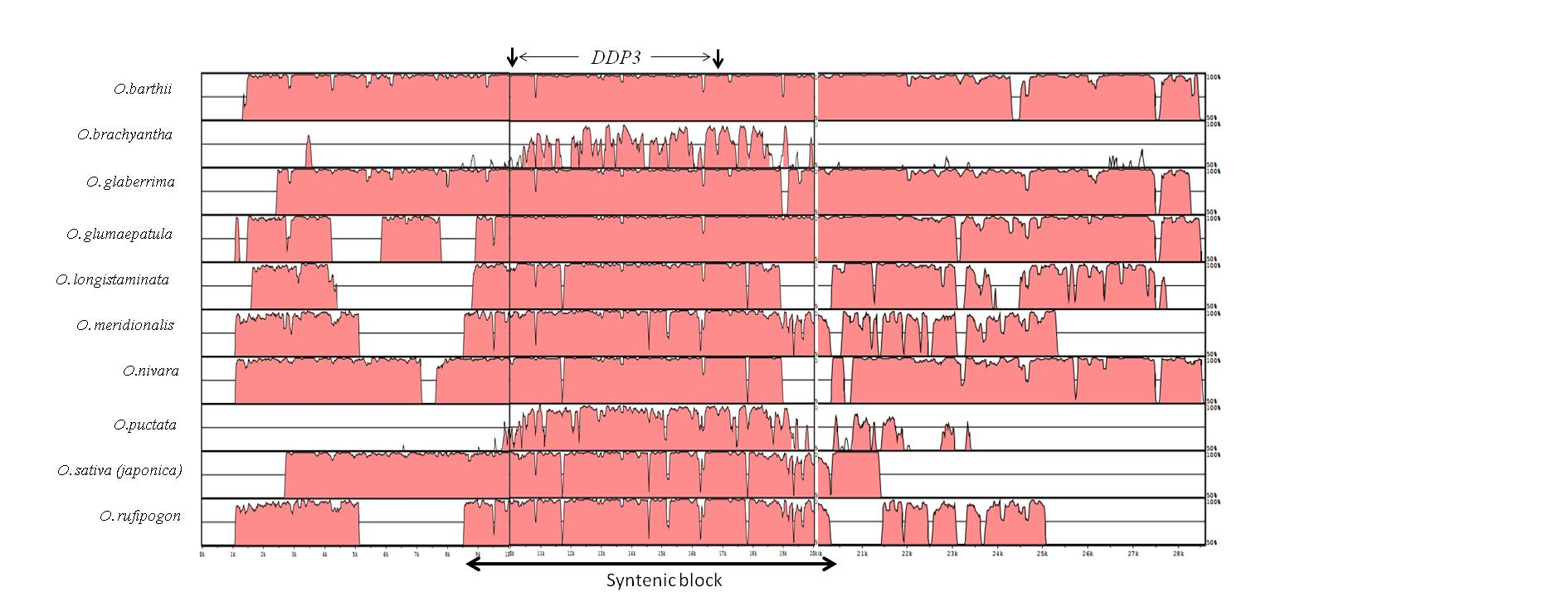

Supplement: S2 Fig — Conserved regions are indicated in pink (90% identity over a 100-bp window). The relative position of the Os-DDP3 and synteny block region is also indicated. (TIFF) [file pone.0182469.s002.tiff]

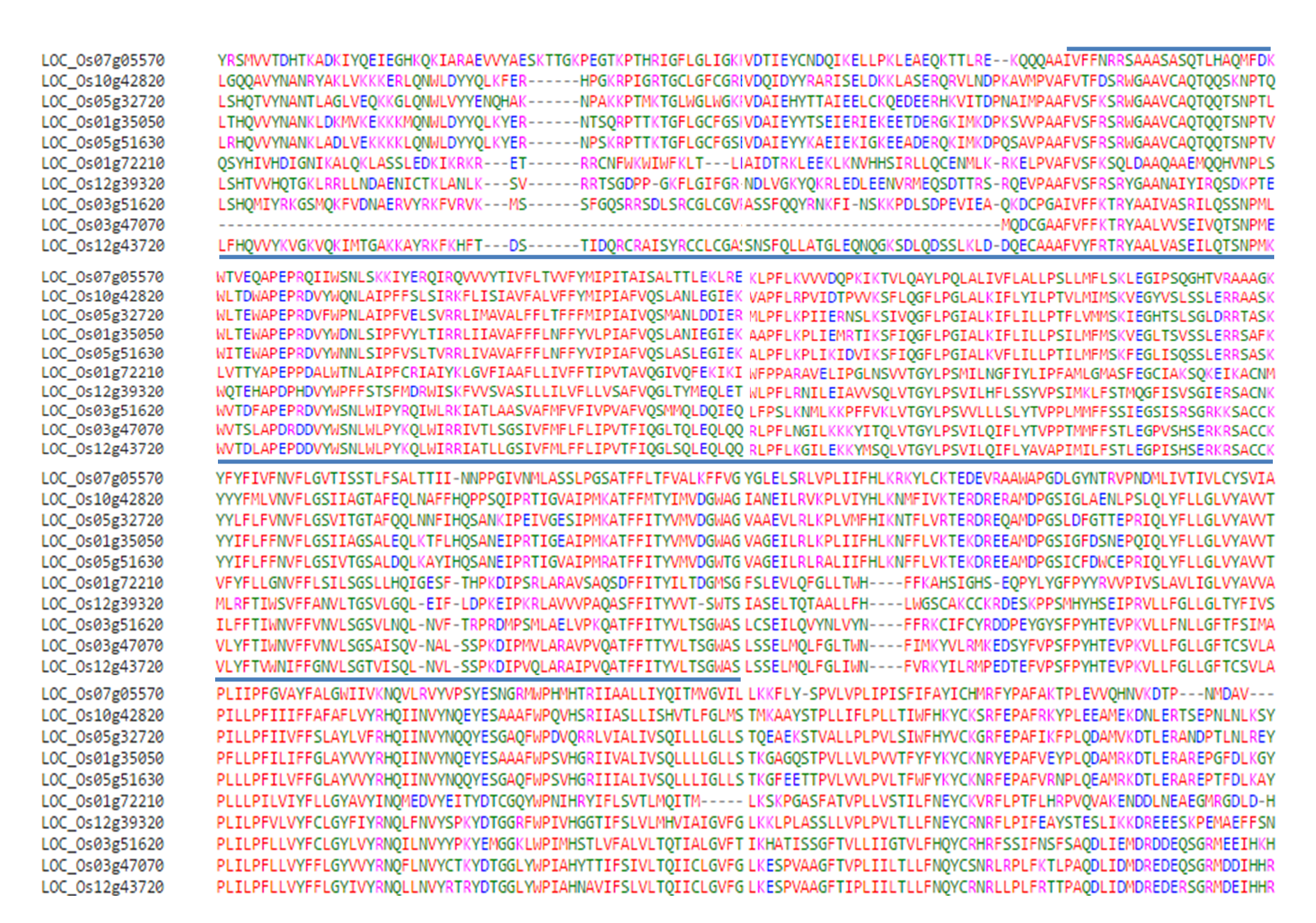

Supplement: S3 Fig — Multiple sequence alignment of DUF221 domain from DDP proteins of rice. The bar line indicates the DUF221 domain. The gene ids can be seen in S2 Table. (TIF) [file pone.0182469.s003.TIF]

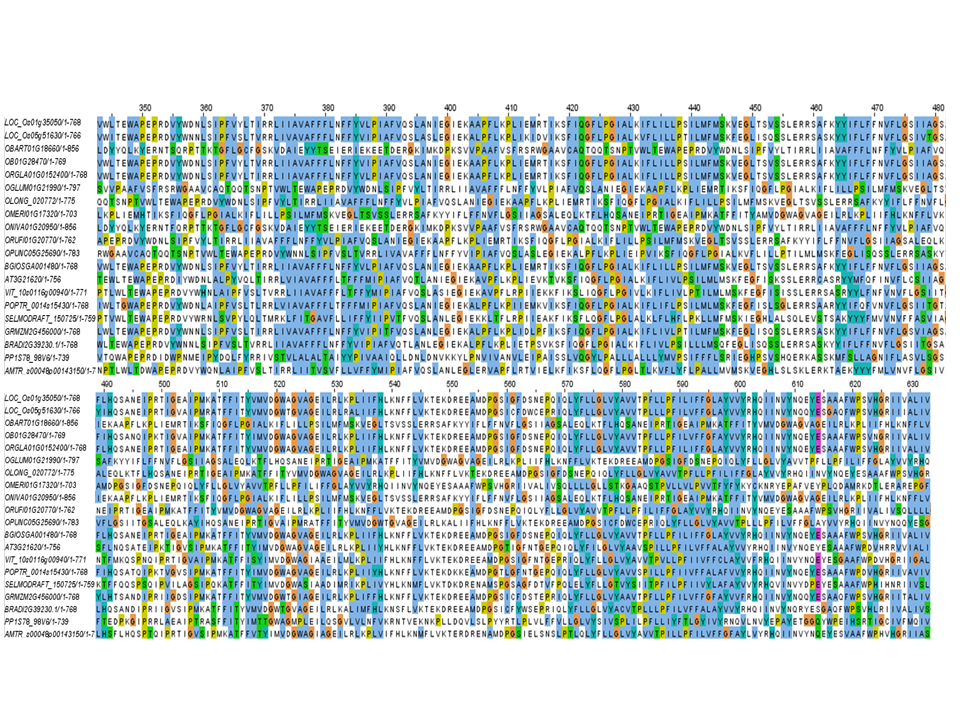

Supplement: S4 Fig — Multiple sequence alignment of DDP proteins of selected species is generated using CLUSTAL X and visualized in Jalview. The gene ids of the different species can be seen in S2 Table. (TIF) [file pone.0182469.s004.TIF]

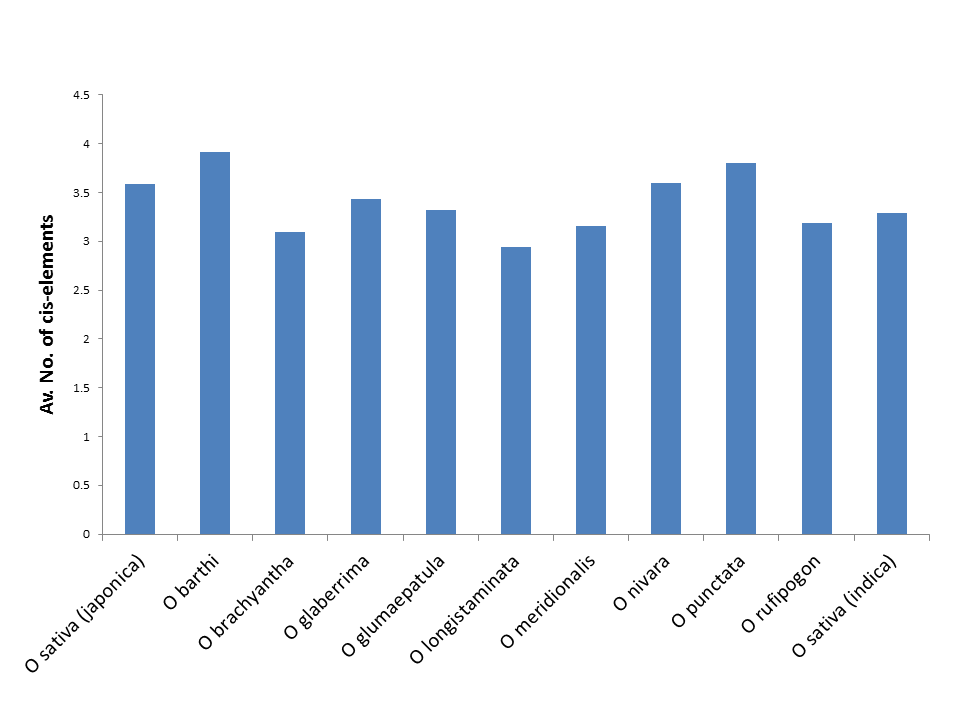

Supplement: S5 Fig — Bar diagram showing that the average number of cis-elements in the 2 kb promoter regions of DDP genes of rice and its wild species as well as O. sativa (indica) is almost uniform. (TIF) [file pone.0182469.s005.TIF]

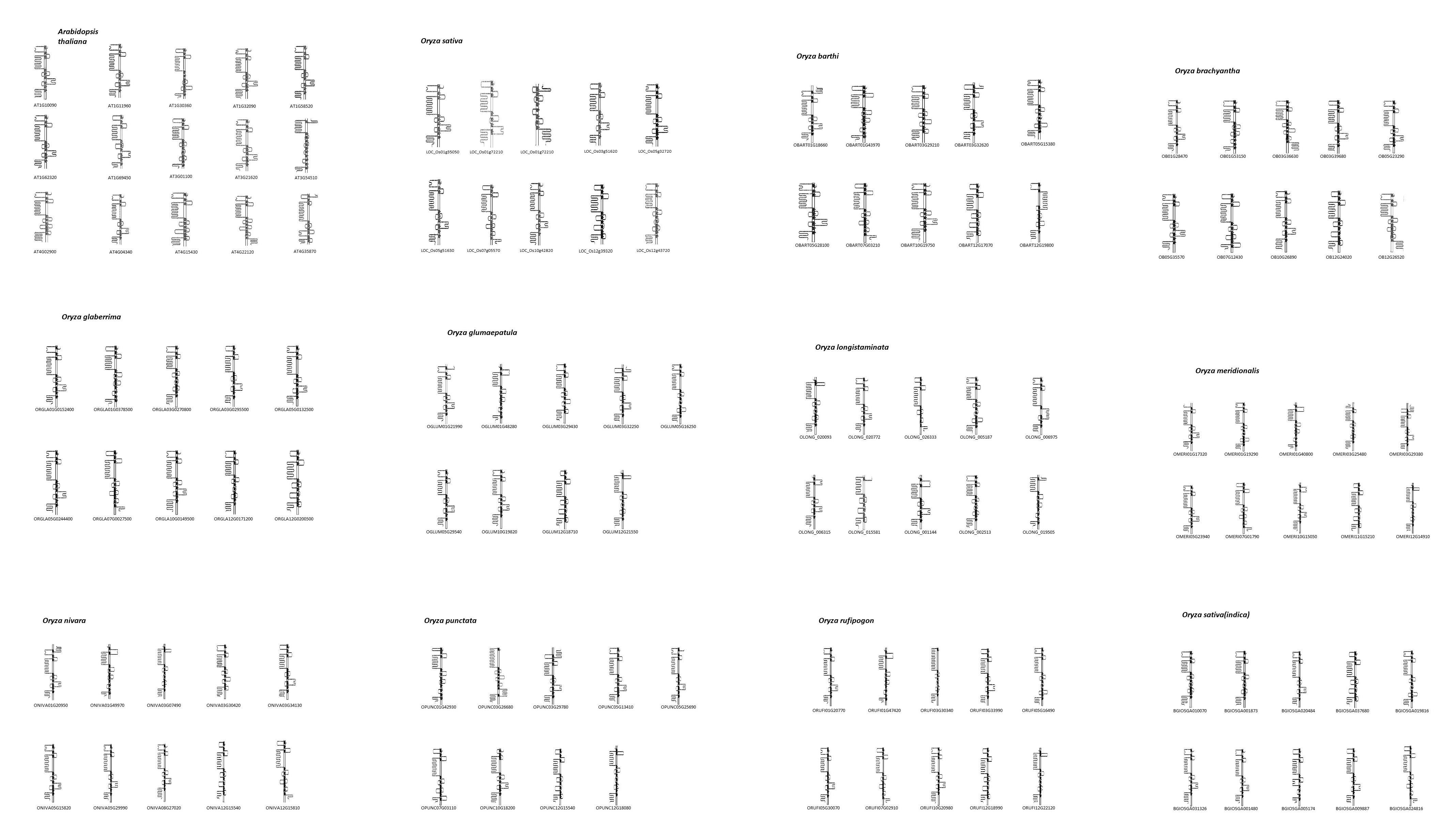

Supplement: S6 Fig — Predicted topological structures of all DDP family members of rice, wild rice and Arabidopsis. Topological structures were predicted using Protter v1.0. For splice variants, topological architecture for only the largest transcript has been provided. Finger-like projections represent loops joining two clusters of TMs. The right side of each structure represents the extra-cellular region while as, left side the intra-cellular region. (JPG) [file pone.0182469.s006.jpg]

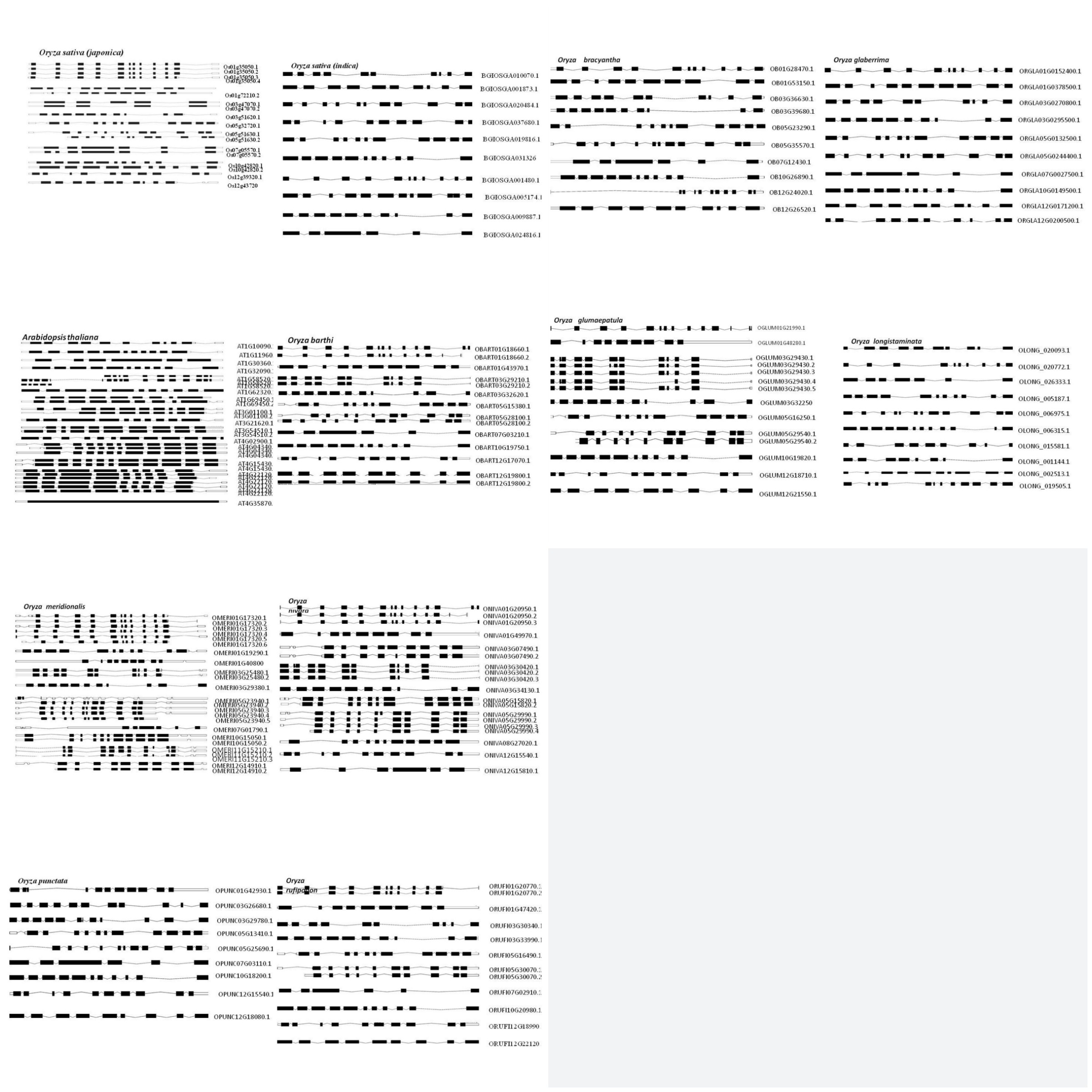

Supplement: S7 Fig — Typical structure of different DDP genes. Lengths are drawn according to the size of genes. Black boxes indicate the coding exons, lines joining them indicate the introns which were not drawn to scale. The white color boxes represent the non-coding exons, whereas the white boxes at the ends represent the UTRs. It can be seen that the average number of introns in DDP gene families of different species was found to be conserved (approximately 9 introns). However, frequent length changes in exons, introns and UTRs can be seen. (TIFF) [file pone.0182469.s007.tiff]

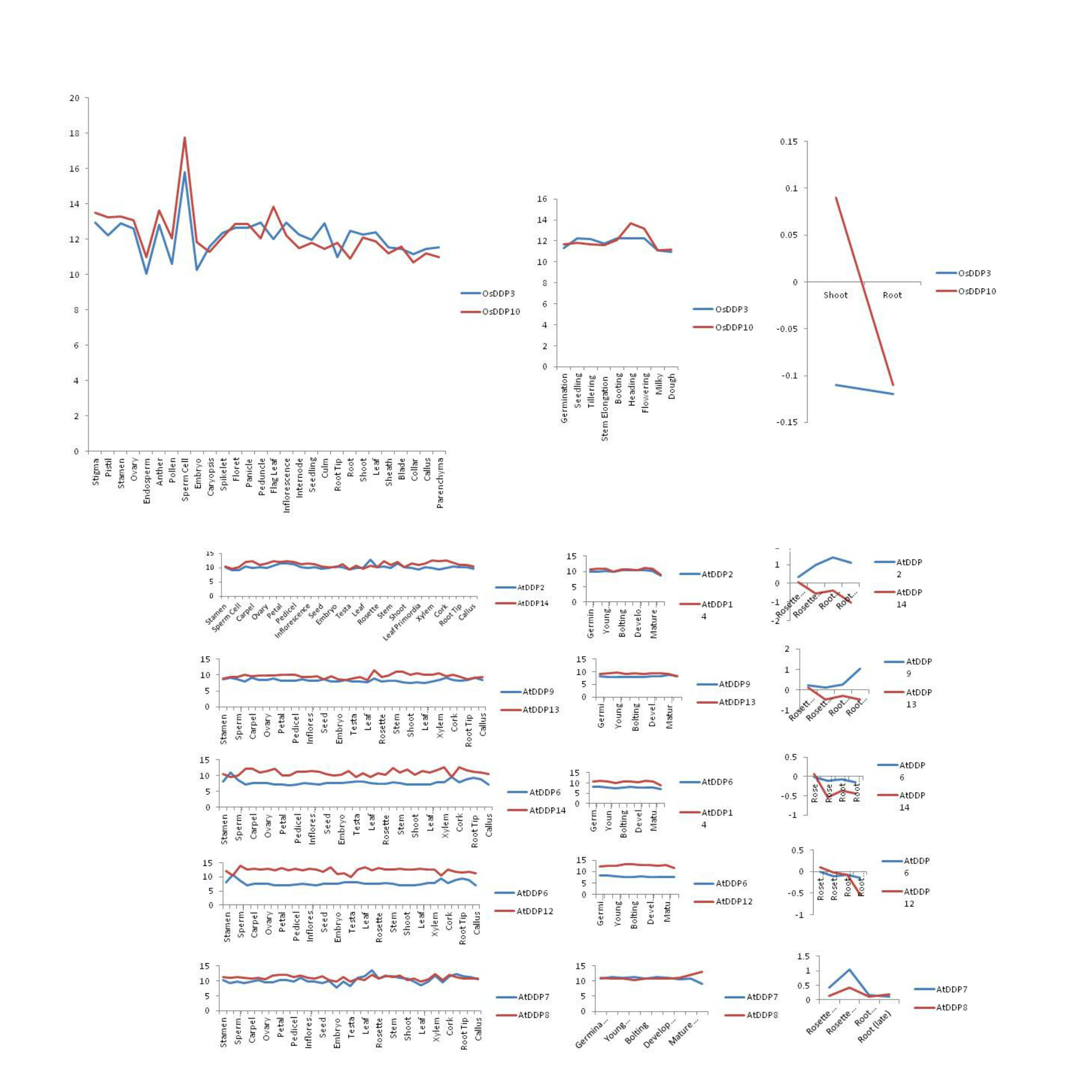

Supplement: S8 Fig — The line graphs represent the microarray-based expression profiles for one and six pairs of duplicated genes in rice (A) and Arabidopsis (B) respectively, under salinity, in different tissues as well as across several developmental stages. The expression profile of every duplicated gene pair is shown in three different line graphs to represent tissues, developmental stages and salinity from left to right. (TIFF) [file pone.0182469.s008.tiff]
