# Supplementary material for: Genome-wide analysis of DUF221 domain-containing gene family in Oryza species and identification of its salinity stress-responsive members in rice
Source: PLoS One. 2017 Aug 28;12(8):e0182469. doi: 10.1371/journal.pone.0182469 (PMC5573286; doi:10.1371/journal.pone.0182469)
Supplement: S1 Table — (DOCX) [file pone.0182469.s009.docx]

| Gene name | Transcript | Locus | Chr | Genomic sequence (bp) | CDS(bp) | Deduced peptides | | | | Localization | NLS/NES | No. of ESTs |
| --- | --- | --- | --- | --- | --- | --- | --- | --- | --- | --- | --- | --- |
|  |  |  |  |  |  | Length (aa) | MW (kDa) | PI | GRAVY |  |  |  |
| ***Oryza sativa*** | | | | | | | | | | | |  |
| *OsDDP1* | OsDDP1.1  OsDDP1.2  OsDDP1.3  OsDDP1.4 | LOC_Os01g35050.1  LOC_Os01g35050.2  LOC_Os01g35050.3  LOC_Os01g35050.4 | 1 | 11019 | 2307  2307  2307  2112 | 768  768  768  703 | 87852.4  87852.4  87852.4  80599.9 | 8.69  8.69  8.69  8.30 | 0.122  0.122  0.122  0.098 | Plasma membrane | -/- | 11 |
| *OsDDP2* | OsDDP2.1  OsDDP2.2 | LOC_Os01g72210.1  LOC_Os01g72210.2 | 1 | 4627 | 2106  1725 | 701  574 | 79344.1  65881.3 | 9.26  9.08 | 0.236  0.204 | Plasma membrane | +/+ | 70 |
| *OsDDP3* | OsDDP3.1  OsDDP3.2 | LOC_Os03g47070.1  LOC_Os03g47070.2 | 3 | 3265 | 1443  1167 | 480  388 | 54232.1  43550.4 | 8.00  7.59 | 0.227  0.104 | Plasma membrane | -/- | 33 |
| *OsDDP4* | OsDDP4.1 | LOC_Os03g51620.1 | 3 | 6292 | 2232 | 743 | 85137.1 | 8.94 | 0.145 | Plasma membrane | -/+ | 10 |
| *OsDDP5* | OsDDP5.1 | LOC_Os05g32720.1 | 5 | 5962 | 2304 | 767 | 87589.1 | 8.81 | 0.133 | Plasma membrane | -/+ | 5 |
| *OsDDP6* | OsDDP6.1  OsDDP6.2 | LOC_Os05g51630.1  LOC_Os05g51630.2 | 5 | 8252 | 2301  2301 | 766  766 | 87926.6  87926.6 | 8.89  8.89 | 0.137  0.137 | Plasma membrane | -/+ | 12 |
| *OsDDP7* | OsDDP7.1  OsDDP7.2 | LOC_Os07g05570.1  LOC_Os07g05570.2 | 7 | 5039 | 2196  2079 | 731  692 | 82114.4  77615.7 | 9.32  9.22 | 0.272  0.214 | Plasma membrane | -/+ | 6 |
| *OsDDP8* | OsDDP8.1  OsDDP8.2 | LOC_Os10g42820.1  LOC_Os10g42820.2 | 10 | 5323 | 2433  2172 | 810  723 | 93829.8  84016.4 | 8.93  8.71 | -0.015  -0.039 | Plasma membrane | -/+ | 73 |
| *OsDDP9* | OsDDP9.1 | LOC_Os12g39320.1 | 12 | 6194 | 2088 | 695 | 78550.4 | 9.00 | 0.223 | Plasma membrane | -/+ | 35 |
| *OsDDP10* | OsDDP10.1 | LOC_Os12g43720.1 | 12 | 6013 | 2292 | 763 | 86888.6 | 9.01 | 0.221 | Plasma membrane | -/+ | 46 |
| **Arabidopsis** | | | | | | | | | | | |  |
| *AtDDP1* | AtDDP1.1 | AT1G10090.1 | 1 | 5888 | 2289 | 762 | 88767.5 | 8.36 | 0.212 | Plasma membrane | -/+ |  |
| *AtDDP2* | AtDDP2.1 | AT1G11960.1 | 1 | 4170 | 2316 | 771 | 88278.9 | 9.14 | 0.120 | Plasma membrane | -/- |  |
| *AtDDP3* | AtDDP3.1 | AT1G30360.1 | 1 | 3333 | 2175 | 724 | 81934.2 | 9.61 | 0.298 | Plasma membrane | -/+ |  |
| *AtDDP4* | AtDDP4.1 | AT1G32090.1 | 1 | 4107 | 2421 | 806 | 93132.4 | 9.36 | 0.031 | Plasma membrane | -/+ |  |
| *AtDDP5* | AtDDP5.1  AtDDP5.2  AtDDP5.3 | AT1G58520.1  AT1G58520.2  AT1G58520.3 | 1 | 8740 | 2241  3126  1029 | 746  1041  342 | 84847.3  117626.7  38105.5 | 8.27  8.51  8.24 | 0.266  0.163  -0.017 | Plasma membrane | -/+ |  |
| *AtDDP6* | AtDDP6.1 | AT1G62320.1 | 1 | 3188 | 2310 | 769 | 88236.0 | 9.35 | 0.076 | Plasma membrane | -/- |  |
| *AtDDP7* | AtDDP7.1  AtDDP7.2 | AT1G69450.1  AT1G69450.2 | 1 | 3029 | 2136  2136 | 711  711 | 81789.7  81789.7 | 9.80  9.80 | 0.285  0.285 | Plasma membrane | +/+ |  |
| *AtDDP8* | AtDDP8.1  AtDDP8.2 | AT3G01100.1  AT3G01100.2 | 3 | 3811 | 2112  1791 | 703  596 | 79725.5  67532.6 | 8.84  8.86 | 0.338  0.410 | Plasma membrane | -/+ |  |
| *AtDDP9* | AtDDP9.1 | AT3G21620.1 | 3 | 3504 | 2271 | 756 | 86685.8 | 9.43 | 0.189 | Plasma membrane | -/- |  |
| *AtDDP10* | AtDDP10.1  AtDDP10.2 | AT3G54510.1  AT3G54510.2 | 3 | 3218 | 1854  2139 | 617  712 | 70653.7  81398.2 | 7.46  8.32 | 0.122  0.141 | Plasma membrane | -/+ |  |
| *AtDDP11* | AtDDP11 | AT4G02900.1 | 4 | 3682 | 2358 | 785 | 89777.9 | 8.52 | 0.141 | Plasma membrane | -/- |  |
| *AtDDP12* | AtDDP12.1  AtDDP12.2  AtDDP12.3 | AT4G04340.1  AT4G04340.2  AT4G04340.3 | 4 | 4160 | 2319  2319  2319 | 772  772  772 | 87606.5  87606.5  87606.5 | 8.76  8.76  8.76 | 0.176  0.176  0.176 | Plasma membrane | -/+ |  |
| *AtDDP13* | AtDDP13.1  AtDDP13.2 | AT4G15430.1  AT4G15430.2 | 4 | 3629 | 2286  2283 | 761  760 | 87166.0  87037.9 | 9.54  9.54 | 0.097  0.101 | Plasma membrane | -/+ |  |
| *AtDDP14* | AtDDP14.1  AtDDP14.2  AtDDP14.3  AtDDP14.4  AtDDP14.5  AtDDP14.6 | AT4G22120.1  AT4G22120.2  AT4G22120.3  AT4G22120.4  AT4G22120.5  AT4G22120.6 | 4 | 3969 | 2316  2316  2316  2316  2316  2316 | 771  771  771  771  771  771 | 87893.0  87893.0  87893.0  87893.0  87893.0  87893.0 | 9.05  9.05  9.05  9.05  9.05  9.05 | 0.211  0.211  0.211  0.211  0.211  0.211 | Plasma membrane | -/- |  |
| *AtDDP15* | AtDDP15.1 | AT4G35870.1 | 4 | 2716 | 2454 | 817 | 92583.8 | 7.37 | 0.216 | Plasma membrane | -/+ |  |
| ***Oryza barthi*** | | | | | | | | | | | |  |
| *ObarDDP1* | ObarDDP1.1 | OBART01G18660 | 1 | 10803 | 2,571 | 856 | 97581.5 | 9.01 | 0.078 | Plasma membrane | +/- |  |
| *ObarDDP2* | ObarDDP2.1 | OBART01G43970 | 1 | 3981 | 2,454 | 720 | 81456.7 | 9.26 | 0.269 | Plasmamembrane | +/+ |  |
| *ObarDDP3* | ObarDDP3.1  ObarDDP3.2 | OBART03G29210.1  OBART03G29210.2 | 3 | 8068 | 2,304  2370 | 755  777 | 85116.3  87683.4 | 9.17  9.11 | 0.200  0.202 | Plasma membrane | -/+ |  |
| *ObarDDP4* | ObarDDP4.1 | OBART03G32620 | 3 | 5857 | 2,364 | 787 | 90068.7 | 9.15 | 0.078 | Plasma membrane | -/+ |  |
| *ObarDDP5* | ObarDDP5.1 | OBART05G15380 | 5 | 5968 | 2,751 | 783 | 89377.3 | 8.86 | 0.151 | Plasma membrane | -/+ |  |
| *ObarDDP6* | ObarDDP6.1  ObarDDP6.2 | OBART05G28100.1  OBART05G28100.2 | 5 | 8304 | 2,789  2724 | 739  739 | 84961.3  84961.3 | 9.12  9.12 | 0.149  0.149 | Plasma membrane | +/+ |  |
| *ObarDDP7* | ObarDDP7.1 | OBART07G03210 | 7 | 4451 | 2196 | 731 | 82076.2 | 9.32 | 0.259 | Plasma membrane | -/+ |  |
| *ObarDDP8* | ObarDDP8.1 | OBART10G19750 | 10 | 4980 | 2,433 | 810 | 93829.9 | 8.98 | -0.013 | Plasma membrane | -/+ |  |
| *ObarDDP9* | ObarDDP9.1 | OBART12G17070 | 12 | 6203 | 2,614 | 695 | 78517.5 | 9.03 | 0.224 | Plasma membrane | -/+ |  |
| *ObarDDP10* | ObarDDP10.1  ObarDDP10.2 | OBART12G19800.1  OBART12G19800.2 | 12 | 4243 | 2216  2312 | 737  769 | 84002.1  87556.4 | 9.02  8.96 | 0.166  0.218 | Plasma membrane | -/- |  |
| ***Oryza brachyantha*** |  |  |  |  |  |  |  |  |  |  |  |  |
| *ObDDP1* | ObDDP1.1 | OB01G28470 | 1 | 8603 | 2661 | 769 | 88014.7 | 8.93 | 0.110 | Plasma membrane | -/- |  |
| *ObDDP2* | ObDDP2.1 | OB01G53150 | 1 | 3842 | 2189 | 714 | 80587.6 | 9.07 | 0.267 | Plasma membrane | -/+ |  |
| *ObDDP3* | ObDDP3.1 | OB03G36630 | 3 | 6692 | 2632 | 779 | 87916.6 | 9.14 | 0.207 | Plasma membrane | -/+ |  |
| *ObDDP4* | ObDDP4.1 | OB03G39680 | 3 | 6752 | 2522 | 743 | 85305.3 | 8.98 | 0.154 | Plasma membrane | -/+ |  |
| *ObDDP5* | ObDDP5.1 | OB05G23290 | 5 | 6474 | 2304 | 767 | 87682.2 | 8.81 | 0.121 | Plasma membrane | -/- |  |
| *ObDDP6* | ObDDP6.1 | OB05G35570 | 5 | 8278 | 3052 | 770 | 88581.5 | 9.11 | 0.136 | Plasma membrane | -/- |  |
| *ObDDP7* | ObDDP7.1 | OB07G12430 | 7 | 4862 | 2807 | 729 | 81668.7 | 9.25 | 0.280 | Plasma membrane | -/+ |  |
| *ObDDP8* | ObDDP8.1 | OB10G26890 | 10 | 5897 | 2880 | 807 | 93452.3 | 9.05 | 0.016 | Plasma membrane | -/+ |  |
| *ObDDP9* | ObDDP9.1 | OB12G24020 | 12 | 6448 | 2687 | 672 | 75896.3 | 8.92 | 0.259 | Plasma membrane | -/- |  |
| *ObDDP10* | ObDDP10.1 | OB12G26520 | 12 | 4406 | 2611 | 764 | 86907.7 | 9.04 | 0.224 | Plasma membrane | -/+ |  |
| ***Oryza glaberrima*** |  |  |  |  |  |  |  |  |  |  |  |  |
| *OglaDDP1* | OglaDDP1.1 | ORGLA01G0152400 | 1 | 8550 | 2307 | 768 | 87852.4 | 8.69 | 0.122 | Plasma membrane | -/- |  |
| *OglaDDP2* | OglaDDP2.1 | ORGLA01G0378500 | 1 | 3689 | 2124 | 707 | 79989.9 | 9.23 | 0.250 | Plasma membrane | +/+ |  |
| *OglaDDP3* | OglaDDP3.1 | ORGLA03G0270800 | 3 | 5983 | 2334 | 777 | 87711.4 | 9.15 | 0.201 | Plasma membrane | -/+ |  |
| *OglaDDP4* | OglaDDP4.1 | ORGLA03G0295500 | 3 | 4809 | 2232 | 743 | 85196.2 | 8.89 | 0.140 | Plasma membrane | -/+ |  |
| *OglaDDP5* | OglaDDP5.1 | ORGLA05G0132500 | 5 | 5217 | 2304 | 767 | 87575.1 | 8.81 | 0.133 | Plasma membrane | -/+ |  |
| *OglaDDP6* | OglaDDP6.1 | ORGLA05G0244400 | 5 | 6503 | 2301 | 766 | 87940.7 | 8.89 | 0.137 | Plasma membrane | -/+ |  |
| *OglaDDP7* | OglaDDP7.1 | ORGLA07G0027500 | 7 | 4450 | 2196 | 731 | 82076.2 | 9.32 | 0.259 | Plasma membrane | -/+ |  |
| *OglaDDP8* | OglaDDP8.1 | ORGLA10G0149500 | 10 | 4979 | 2433 | 810 | 93829.9 | 8.98 | -0.013 | Plasma membrane | -/+ |  |
| *OglaDDP9* | OglaDDP9.1 | ORGLA12G0171200 | 12 | 4242 | 2292 | 763 | 87011.7 | 9.07 | 0.214 | Plasma membrane | -/+ |  |
| *OglaDDP10* | OglaDDP10.1 | ORGLA12G0200500 | 12 | 5677 | 2088 | 695 | 78607.5 | 9.05 | 0.215 | Plasma membrane | -/+ |  |
|  |  |  |  |  |  |  |  |  |  |  |  |  |
| ***Oryza glumaepatula*** | | | | | | | | | | | |  |
| *OglDDP1* | OglDDP1.1 | OGLUM01G21990 | 1 | 10852 | 2394 | 797 | 91065.3 | 8.77 | 0.123 | Plasma membrane | -/- |  |
| *OglDDP2* | OglDDP2.1 | OGLUM01G48280 | 1 | 5763 | 4054 | 659 | 74807.9 | 9.16 | 0.262 | Plasma membrane | -/+ |  |
| *OglDDP3* | OglDDP3.1  OglDDP3.2  OglDDP3.3  OglDDP3.4  OglDDP3.5 | OGLUM03G29430.1  OGLUM03G29430.2  OGLUM03G29430.3  OGLUM03G29430.4  OGLUM03G29430.5 | 3 | 8046 | 2304  2232  2370  1986  2424 | 755  731  777  649  777 | 85206.4  82231.8  87773.4  73329.1  87773.4 | 9.17  9.04  9.11  8.87  9.11 | 0.196  0.162  0.198  0.122  0.198 | Plasma membrane | -/+ |  |
| *OglDDP4* | OglDDP4.1 | OGLUM03G32250 | 3 | 5900 | 2397 | 798 | 90965.7 | 9.20 | 0.061 | Plasma membrane | -/+ |  |
| *OglDDP5* | OglDDP5.1 | OGLUM05G16250 | 5 | 6026 | 2808 | 783 | 89451.3 | 8.89 | 0.147 | Plasma membrane | -/+ |  |
| *OglDDP6* | OglDDP6.1  OglDDP6.2 | OGLUM05G29540.1  OGLUM05G29540.2 | 5 | 8394 | 2986  2721 | 739  731 | 84848.1  84848.1 | 9.07  9.07 | 0.154  0.154 | Plasma membrane | +/+ |  |
| *OglDDP7* | OglDDP7.1 | OGLUM10G19820 | 10 | 4987 | 2442 | 813 | 94185.2 | 8.92 | -0.026 | Plasma membrane | -/+ |  |
| *OglDDP8* | OglDDP8.1 | OGLUM12G18710 | 12 | 6142 | 2554 | 682 | 76990.6 | 8.95 | 0.226 | Plasma membrane | -/+ |  |
| *OglDDP9* | OglDDP9.1 | OGLUM12G21550 | 12 | 4265 | 2216 | 737 | 83872.9 | 8.97 | 0.175 | Plasma membrane | -/- |  |
| ***Oryza longistaminata*** | | | | | | | | | | | |  |
| *OloDDP1* | OloDDP1.1 | OLONG_020093 |  | 4424 | 2163 | 737 | 83858.8 | 8.93 | 0.176 | Plasma membrane | -/+ |  |
| *OloDDP2* | OloDDP2.1 | OLONG_020772 |  | 9301 | 2328 | 775 | 88685.4 | 8.59 | 0.125 | Plasma membrane | -/- |  |
| *OloDDP3* | OloDDP3.1 | OLONG_026333 |  | 4157 | 1971 | 656 | 73595.7 | 9.16 | 0.153 | Plasma membrane | -/+ |  |
| *OloDDP4* | OloDDP4.1 | OLONG_005187 |  | 5455 | 2352 | 743 | 84852.0 | 8.43 | 0.189 | Plasma membrane | -/+ |  |
| *OloDDP5* | OloDDP5.1 | OLONG_006975 |  | 6033 | 2355 | 703 | 80608.9 | 8.68 | 0.087 | Plasma membrane | -/+ |  |
| *OloDDP6* | OloDDP6.1 | OLONG_006315 |  | 4967 | 2520 | 770 | 88766.6 | 8.70 | -0.070 | Plasma membrane | -/+ |  |
| *OloDDP7* | OloDDP7.1 | OLONG_015581 |  | 5944 | 1806 | 596 | 67865.7 | 8.63 | 0.128 | Plasma membrane | -/+ |  |
| *OloDDP8* | OloDDP8.1 | OLONG_001144 |  | 4822 | 1689 | 694 | 79816.9 | 8.93 | 0.129 | Plasma membrane | -/+ |  |
| *OloDDP9* | OloDDP9.1 | OLONG_002513 |  | 8018 | 1716 | 777 | 87797.5 | 9.11 | 0.197 | Plasma membrane | -/+ |  |
| *OloDDP10* | OloDDP10.1 | OLONG_019505 |  | 3936 | 2235 | 618 | 70438.7 | 9.06 | 0.236 | Plasma membrane | -/+ |  |
| ***Oryza meridionalis*** | | | | | | | | | | | |  |
| *OmeDDP1* | OmeDDP1.1  OmeDDP1.2  OmeDDP1.3  OmeDDP1.4  OmeDDP1.5  OmeDDP1.6 | OMERI01G17320.1  OMERI01G17320.2  OMERI01G17320.3  OMERI01G17320.4  OMERI01G17320.5  OMERI01G17320.6 | 1 | 12146 | 3194  3574  3565  2908  2887  3123 | 703  762  768  793  786  768 | 80554.8  87291.8  87807.3  90539.3  89742.4  87807.3 | 8.04  8.69  8.59  8.74  8.74  8.59 | 0.093  0.123  0.117  0.089  0.086  0.117 | Plasma membrane | -/+ |  |
| *OmeDDP2* | OmeDDP2.1 | OMERI01G19290 | 1 | 6058 | 2972 | 783 | 89438.2 | 8.89 | 0.136 | Plasma membrane | -/+ |  |
| *OmeDDP3* | OmeDDP3.1 | OMERI01G40800 | 1 | 8611 | 6849 | 660 | 74865.9 | 9.05 | 0.268 | Plasma membrane | -/+ |  |
| *OmeDDP4* | OmeDDP4.1  OmeDDP4.2 | OMERI03G25480.1  OMERI03G25480.2 | 3 | 10333 | 3518  3625 | 744  777 | 84283.2  87825.5 | 9.03  9.11 | 0.168  0.196 | Plasma membrane | -/- |  |
| *OmeDDP5* | OmeDDP5.1 | OMERI03G29380 | 3 | 6727 | 2911 | 843 | 96091.6 | 9.52 | -0.007 | Plasma membrane | -/+ |  |
| *OmeDDP6* | OmeDDP6.1  OmeDDP6.2  OmeDDP6.3  OmeDDP6.4  OmeDDP6.5  OmeDDP6.6 | OMERI05G23940.1  OMERI05G23940.2  OMERI05G23940.3  OMERI05G23940.4  OMERI05G23940.5  OMERI05G23940.6 | 5 | 11390 | 5834  5755  5658  5619  3448  267 | 90  766  779  766  766  88 | 87816.5  87816.5  89347.4  87816.5  87816.5  9994.3 | 8.83  8.83  8.86  8.83  8.83  6.04 | 0.142  0.142  0.157  0.142  0.142  -0.235 | Plasma membrane | -/+ |  |
| *OmeDDP7* | OmeDDP7.1 | OMERI07G01790 | 7 | 10937 | 6397 | 731 | 82050.1 | 9.29 | 0.253 | Plasma membrane | -/+ |  |
| *OmeDDP8* | OmeDDP8.1  OmeDDP8.2 | OMERI10G15050.1  OMERI10G15050.2 | 10 | 6869 | 3846  3956 | 788  788 | 90912.2  90912.2 | 8.91  8.91 | -0.048  -0.048 | Plasma membrane | -/+ |  |
| *OmeDDP9* | OmeDDP9.1  OmeDDP9.2  OmeDDP9.3 | OMERI11G15210.1  OMERI11G15210.2  OMERI11G15210.3 | 11 | 10254 | 3007  2821  2692 | 690  628  585 | 77982.8  70953.6  65883.8 | 9.00  9.18  9.20 | 0.215  0.201  0.195 | Plasma membrane | -/+ |  |
| *OmeDDP10* | OmeDDP10.1  OmeDDP10.2 | OMERI12G14910.1  OMERI12G14910.2 | 12 | 5955 | 2981  2651 | 737  737 | 83949.0  83949.0 | 8.97  8.97 | 0.169  0.169 | Plasma membrane | -/- |  |
| ***Oryza nivara*** | | | | | | | | | | | |  |
| *OniDDP1* | OniDDP1.1  OniDDP1.2  OniDDP1.3 | ONIVA01G20950.1  ONIVA01G20950.2  ONIVA01G20950.3 | 1 | 11786 | 2571  2349  2424 | 856  782  807 | 97440.4  89329.3  92209.6 | 8.98  8.55  9.09 | 0.095  0.141  0.107 | Plasma membrane | +/- |  |
| *OniDDP2* | OniDDP2.1 | ONIVA01G49970 | 1 | 4857 | 3147 | 658 | 74607.7 | 9.24 | 0.265 | Plasma membrane | -/+ |  |
| *OniDDP3* | OniDDP3.1  OniDDP3.2 | ONIVA03G07490.1  ONIVA03G07490.2 | 3 | 6124 | 3183  2934 | 737  654 | 83858.9  74230.5 | 8.97  9.03 | 0.175  0.139 | Plasma membrane | -/- |  |
| *OniDDP4* | OniDDP4.1  OniDDP4.2  OniDDP4.3 | ONIVA03G30420.1  ONIVA03G30420.2  ONIVA03G30420.3 | 3 | 8047 | 2223  2370  2223 | 755  777  728 | 85216.4  87783.5  82258.0 | 9.17  9.11  9.13 | 0.195  0.197  0.173 | Plasma membrane | -/+ |  |
| *OniDDP5* | OniDDP5.1 | ONIVA03G34130 | 3 | 4811 | 2232 | 743 | 85193.2 | 8.89 | 0.139 | Plasma membrane | -/+ |  |
| *OniDDP6* | OniDDP6.1  OniDDP6.2 | ONIVA05G15820.1  ONIVA05G15820.2 | 5 | 6119 | 2825  2805 | 767  767 | 87601.2  87601.2 | 8.81  8.81 | 0.140  0.140 | Plasma membrane | -/+ |  |
| *OniDDP7* | OniDDP7.1  OniDDP7.2  OniDDP7.3  OniDDP7.4 | ONIVA05G29990.1  ONIVA05G29990.2  ONIVA05G29990.3  ONIVA05G29990.4 | 5 | 8369 | 3115  2955  2853  2894 | 766  739  739  739 | 87940.7  84961.3  84961.3  84961.3 | 8.89  9.12  9.12  9.12 | 0.137  0.149  0.149  0.149 | Plasma membrane | -/+ |  |
| *OniDDP8* | OniDDP8.1 | ONIVA08G27020 | 8 | 5798 | 3257 | 812 | 94010.0 | 8.86 | -0.018 | Plasma membrane | -/+ |  |
| *OniDDP9* | OniDDP9.1 | ONIVA12G15540 | 12 | 6334 | 2746 | 695 | 78563.5 | 9.00 | 0.220 | Plasma membrane | -/+ |  |
| *OniDDP10* | OniDDP10.1 | ONIVA12G15810 | 12 | 5073 | 2824 | 731 | 82071.2 | 9.25 | 0.254 | Plasma membrane | -/+ |  |
| ***Oryza punctata*** | | | | | | | | | | | |  |
| *OpuDDP1* | OpuDDP1.1 | OPUNC01G42930 | 1 | 6460 | 3311 | 716 | 81032.1 | 9.15 | 0.262 | Plasma membrane | +/+ |  |
| *OpuDDP2* | OpuDDP2.1 | OPUNC03G26680 | 3 | 6852 | 2670 | 889 | 99687.5 | 9.06 | 0.103 | Plasma membrane | -/- |  |
| *OpuDDP3* | OpuDDP3.1 | OPUNC03G29780 | 3 | 6790 | 2526 | 841 | 95835.9 | 9.29 | -0.037 | Plasma membrane | -/+ |  |
| *OpuDDP4* | OpuDDP4.1 | OPUNC05G13410 | 5 | 5988 | 2797 | 767 | 87611.1 | 8.79 | 0.122 | Plasma membrane | -/+ |  |
| *OpuDDP5* | OpuDDP5.1 | OPUNC05G25690 | 5 | 8389 | 2619 | 783 | 89814.1 | 9.17 | 0.129 | Plasma membrane | -/+ |  |
| *OpuDDP6* | OpuDDP6.1 | OPUNC07G03110 | 7 | 3926 | 2196 | 731 | 82052.3 | 9.32 | 0.281 | Plasma membrane | -/+ |  |
| *OpuDDP7* | OpuDDP7.1 | OPUNC10G18200 | 10 | 5198 | 2340 | 779 | 89687.9 | 9.03 | -0.006 | Plasma membrane | -/+ |  |
| *OpuDDP8* | OpuDDP8.1 | OPUNC12G15540 | 12 | 6199 | 2665 | 695 | 78542.3 | 8.71 | 0.235 | Plasma membrane | -/+ |  |
| *OpuDDP9* | OpuDDP9.1 | OPUNC12G18080 | 12 | 4234 | 2103 | 700 | 79587.1 | 8.71 | 0.228 | Plasma membrane | -/+ |  |
|  |  |  |  |  |  |  |  |  |  |  |  |  |
| ***Oryza rufipogon*** | | | | | | | | | | | |  |
| *OruDDP1* | OruDDP1.1  OruDDP1.2 | ORUFI01G20770.1  ORUFI01G20770.2 | 1 | 10434 | 2289  2307 | 762  768 | 87336.9  87852.4 | 8.78  8.69 | 0.127  0.122 | Plasma membrane | -/- |  |
| *OruDDP2* | OruDDP2.1 | ORUFI01G47420 | 1 | 4491 | 2781 | 658 | 74637.7 | 9.24 | 0.261 | Plasma membrane | -/+ |  |
| *OruDDP3* | OruDDP3.1 | ORUFI03G30340 | 3 | 6873 | 2301 | 766 | 86730.9 | 9.11 | 0.126 | Plasma membrane | -/+ |  |
| *OruDDP4* | OruDDP4.1 | ORUFI03G33990 | 3 | 4819 | 2241 | 743 | 85106.0 | 8.84 | 0.146 | Plasma membrane | -/+ |  |
| *OruDDP5* | OruDDP5.1 | ORUFI05G16490 | 5 | 6098 | 2886 | 783 | 89451.3 | 8.89 | 0.147 | Plasma membrane | -/+ |  |
| *OruDDP6* | OruDDP6.1  OruDDP6.2 | ORUFI05G30070.1  ORUFI05G30070.2 | 5 | 8339 | 2940  3084 | 739  739 | 84947.3  84947.3 | 9.12  9.12 | 0.150  0.150 | Plasma membrane | +/+ |  |
| *OruDDP7* | OruDDP7.1 | ORUFI07G02910 | 7 | 5595 | 2196 | 731 | 82114.4 | 9.32 | 0.272 | Plasma membrane | -/+ |  |
| *OruDDP8* | OruDDP8.1 | ORUFI10G20980 | 10 | 4980 | 2436 | 811 | 93929.0 | 8.92 | -0.017 | Plasma membrane | -/+ |  |
| *OruDDP9* | OruDDP9.1 | ORUFI12G18990 | 12 | 6204 | 2615 | 695 | 78550.4 | 9.00 | 0.223 | Plasma membrane | -/+ |  |
| *OruDDP10* | OruDDP10.1 | ORUFI12G22120 | 12 | 4241 | 2216 | 737 | 83858.9 | 8.97 | 0.175 | Plasma membrane | -/- |  |
| ***Oryza sativa (indica)*** | | | | | | | | | | | |  |
| *OsiDDP1* | OsiDDP1.1  OsiDDP1.2 | BGIOSGA010070 | 3 | 7212 | 2334 | 777 | 87853.6 | 9.11 | 0.199 | Plasma membrane | -/+ |  |
| *OsiDDP2* | OsiDDP2.1 | BGIOSGA001873 | 1 | 4239 | 2292 | 763 | 86888.6 | 9.01 | 0.221 | Plasma membrane | -/+ |  |
| *OsiDDP3* | OsiDDP3.1 | BGIOSGA020484 | 5 | 6378 | 2301 | 766 | 87926.6 | 8.89 | 0.137 | Plasma membrane | -/+ |  |
| *OsiDDP4* | OsiDDP4.1 | BGIOSGA037680 | 12 | 5676 | 2088 | 695 | 78522.4 | 9.00 | 0.223 | Plasma membrane | -/+ |  |
| *OsiDDP5* | OsiDDP5.1 | BGIOSGA019816 | 5 | 5215 | 2352 | 783 | 89451.3 | 8.89 | 0.147 | Plasma membrane | -/+ |  |
| *OsiDDP6* | OsiDDP6.1 | BGIOSGA031326 | 10 | 4978 | 2436 | 811 | 93929.0 | 8.92 | -0.017 | Plasma membrane | -/+ |  |
| *OsiDDP7* | OsiDDP7.1 | BGIOSGA001480 | 1 | 8426 | 2307 | 768 | 87852.4 | 8.69 | 0.122 | Plasma membrane | -/- |  |
| *OsiDDP8* | OsiDDP8.1 | BGIOSGA005174 | 1 | 3687 | 2040 | 679 | 76849.1 | 9.20 | 0.205 | Plasma membrane | +/+ |  |
| *OsiDDP9* | OsiDDP9.1 | BGIOSGA009887 | 3 | 4810 | 2232 | 743 | 85193.2 | 8.89 | 0.139 | Plasma membrane | -/+ |  |
| *OsiDDP10* | OsiDDP10.1 | BGIOSGA024816 | 7 | 4446 | 2196 | 731 | 82060.2 | 9.32 | 0.262 | Plasma membrane | -/+ |  |
