# Supplementary material for: Genome-wide analysis of DUF221 domain-containing gene family in Oryza species and identification of its salinity stress-responsive members in rice
Source: PLoS One. 2017 Aug 28;12(8):e0182469. doi: 10.1371/journal.pone.0182469 (PMC5573286; doi:10.1371/journal.pone.0182469)
Supplement: S9 Table — The sequence logos were generated using WebLogo. (DOCX) [file pone.0182469.s017.DOCX]

**Supplementary Table 9.** Putative motifs identified from OsDDP proteins using MEME. The sequence logos were generated using WebLogo.

| **Table.Putative motifs identified from OsDDP proteins using MEME** | | |
| --- | --- | --- |
| **Motif name** | **Sequence logo** | **E-value** |
| **Motif 1** | 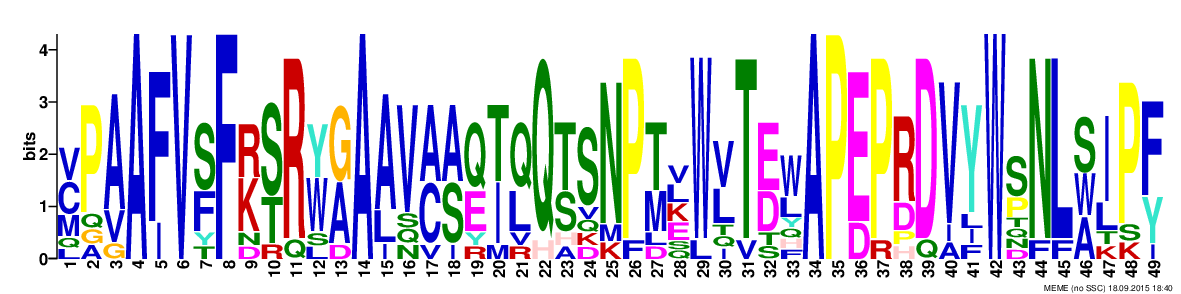  CPAAFVSFKSRWGAAVCAQTQQTSNPTKWVTEWAPEPRDVYWSNLWIPF | 6.3e-200 |
| **Motif 2** | 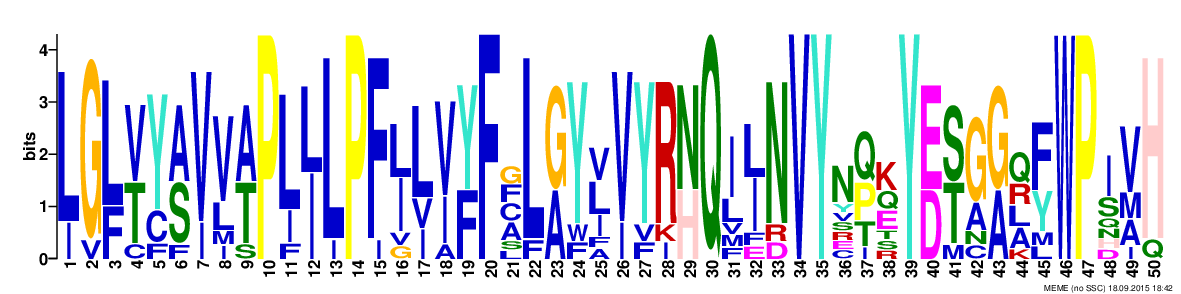  LGLTYAVVTPLILPFILVYFCLGYVVYRNQIINVYNQKYESGGQFWPIVH | 1.2e-188 |
| **Motif 3** | 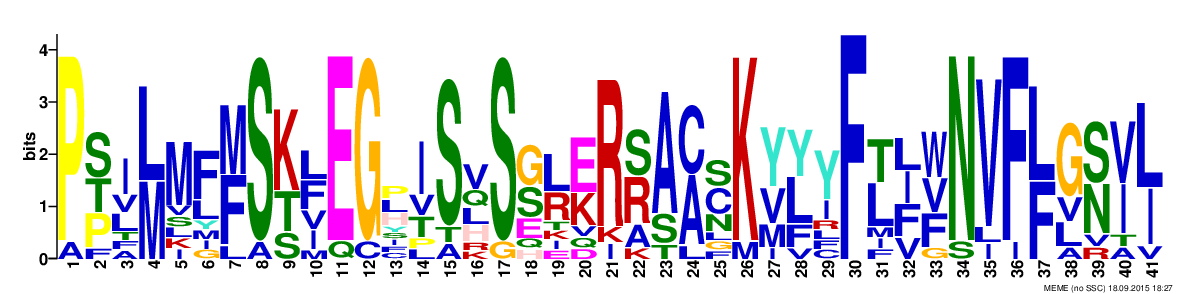  PSIMMFMSKMEGHISHSGRERRACCKYYYFTIWNVFFGNVI | 2.3e-111 |
| **Motif 4** | 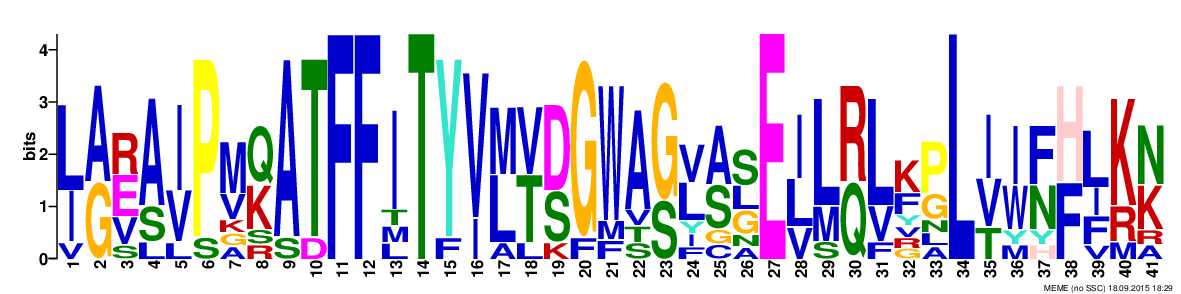  LGEAIPMQATFFITYVMTDGWAGVASEILQLKPLIWNHLKN | 2.3e-102 |
| **Motif 5** | 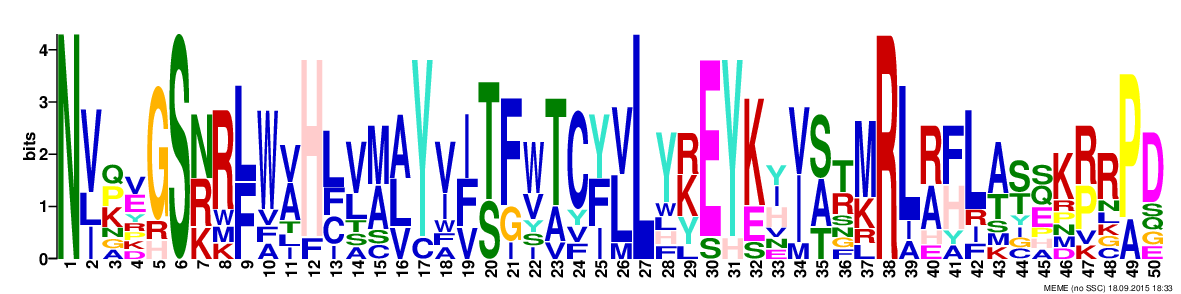  NVQEGSNRFWVHCVMAYVITFWTCYVLYKEYKHISTMRLRHLASQKRRPD | 1.2e-108 |
| **Motif 6** | 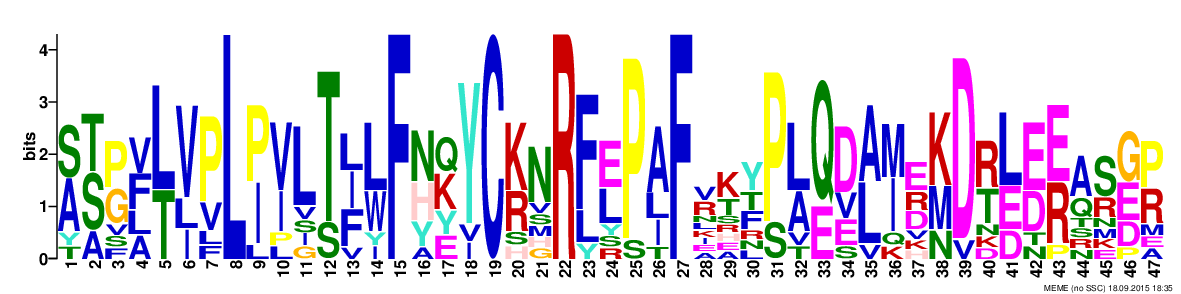  ATPFTVPLPVLTIWFHQYCKNRFEPAFRKYPLQDAMEMDREEEASGP | 6.1e-111 |
| **Motif 7** | 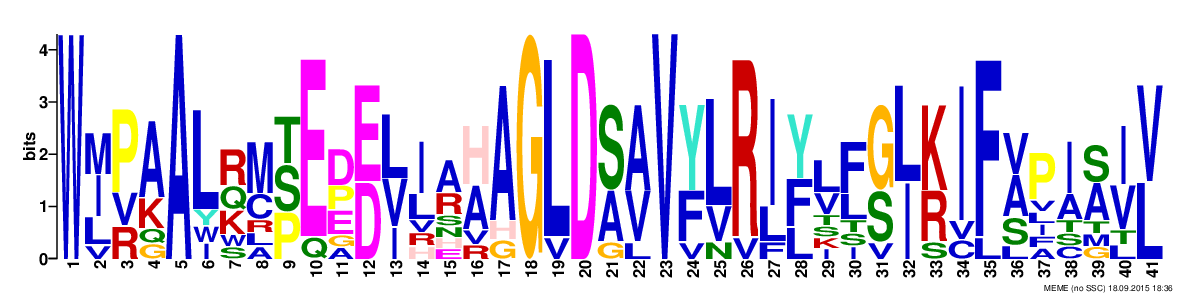  WMPAALRMTEDEVIAHAGLDSAVYLRIYVFGIKIFAPCAIV | 1.8e-080 |
| **Motif 8** | 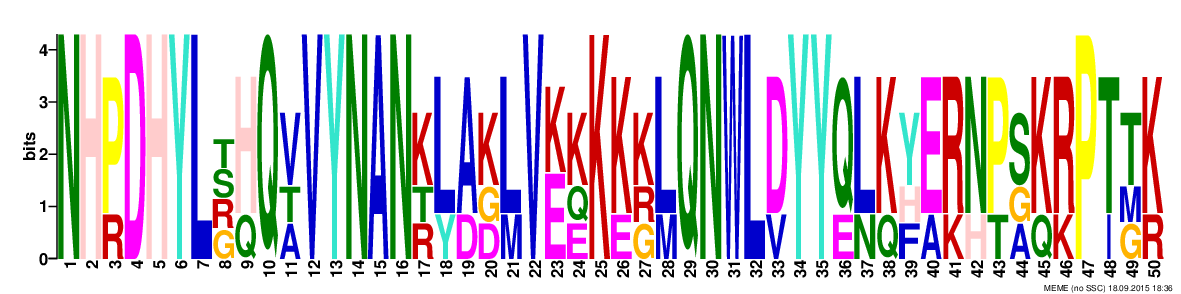  NHPDHYLSHQVVYNANKLAKLVEKKKKLQNWLDYYQLKYERNPSKRPTTK | 9.5e-067 |
| **Motif 9** | 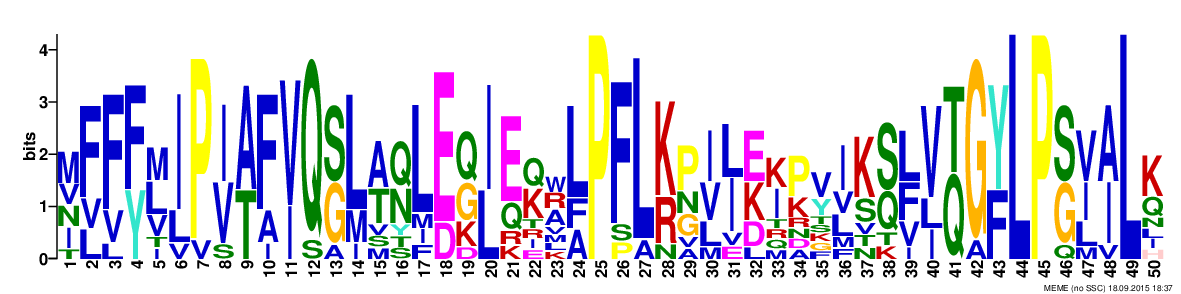  MFFYMIPIAFVQGLAQLEQIEQWLPFLKPIIEKPYIKSFVQGYLPGIALK | 6.8e-109 |
| **Motif 10** | 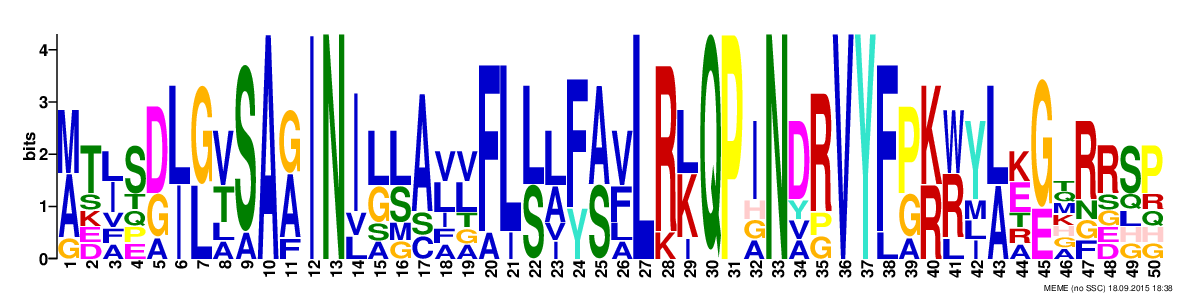  MTISDIGVSAGINIGMAVVFLSLFAVLRKQPINDRVYFPKWYAKGHRRSP | 4.4e-069 |
