# Supplementary material for: Genome-wide analysis of DUF221 domain-containing gene family in Oryza species and identification of its salinity stress-responsive members in rice
Source: PLoS One. 2017 Aug 28;12(8):e0182469. doi: 10.1371/journal.pone.0182469 (PMC5573286; doi:10.1371/journal.pone.0182469)
Supplement: S10 Table — (DOCX) [file pone.0182469.s018.DOCX]

**Supplementary Table 10.** Similarity of motifs identified by MEME analysis in 10 OsDDPs with the known protein domains as analysed by HHPred analysis.

| **Motif** | **Hit (PDB id)** | **Prob.** | **E-value** | **P-value** | **Score** |
| --- | --- | --- | --- | --- | --- |
| **Motif 1** | U1 small nuclear ribonucleoprotein A (2a3j_A) | 97.7 | 5.7E-05 | 1.6E-09 | 40 |
|  | RNA-binding protein 12 (2cqp_A) | 97.59 | 0.00023 | 6.3E-09 | 35.51 |
|  | Insulin-like growth factor 2 mRNA binding protein 3(2e44_A) | 97.5 | 0.00041 | 1.1E-08 | 34.6 |
| **Motif 2** | UPF0092 membrane protein YAJC; drug resistance (2rdd_B) | 70.4 | 3.7 | 0.0001 | 19.2 |
|  | FST, putative uncharacterized protein RNAI (2kv5_A) | 40.2 | 26 | 0.00073 | 16.2 |
|  | PSII-K, photosystem II reaction center protein K | 30.7 | 29 | 0.00082 | 16.5 |
| **Motif 3** | Predicted membrane protein, calcium activation (4wis_A) | 81.6 | 0.95 | 2.7E-05 | 31.7 |
|  | Designed protein; HUB-linked, RHH1 superfamily (2mdv_A) | 59.4 | 11 | 0.00031 | 15.8 |
|  | REPA protein, transcriptional repressor COPG (2cpg_A) | 35.4 | 36 | 0.001 | 14.6 |
| **Motif 4** | Navms, ION transport protein; sodium channel (4cbc_A) | 23.0 | 88 | 0.0025 | 17 |
|  | Sodium channel, ION transport protein (4lto_A) | 22.9 | 88 | 0.0025 | 17.5 |
|  | YSCM2; chaperone, type III secretion (1ttw_B) | 22.2 | 23 | 0.00064 | 17.8 |
| **Motif 5** | VPU protein, U ORF protein (1pi7_A) | 90.1 | 0.68 | 1.9E-05 | 20.7 |
|  | Integrin beta-3; transmembrane signaling (2knc_B) | 65.2 | 14 | 0.00039 | 18.8 |
|  | Sarcolipin; P-type ATPase, hydrolase, calcium transport (3w5a_C) | 61.5 | 10 | 0.00029 | 16.1 |
| **Motif 6** | Hypothetical protein FLJ21157; RNA polymerase II carboxyl- terminal domain (1uzc_A) | 69.6 | 2.4 | 6.7E-05 | 22.8 |
|  | PRE-mRNA processing protein PRP40 (2b7e_A) | 68.4 | 2.3 | 6.5E-05 | 22.3 |
|  | Transcription elongation regulator 1; FF domain (2dod_A) | 58.3 | 5.5 | 0.00015 | 21.9 |
| **Motif 7** | Epidermal growth factor receptor kinase substrate 8 (2e8m_A) | 74.6 | 2.9 | 8E-05 | 22.4 |
|  | 50S ribosomal protein L28; protein biosynthesis (4tp9_X) | 60.9 | 3.7 | 0.0001 | 20.8 |
|  | Hypothetical protein FLJ21935;protein regulation (1wwu_A) | 56.9 | 12 | 0.00033 | 20.0 |
| **Motif 8** | CC-PENT; alpha-helical barrel, coiled coil (4pn8_A) | 66.6 | 8.9 | 0.00025 | 17.1 |
|  | Conserved hypothetical protein; PSI (1ybx_A) | 28.4 | 56 | 0.0016 | 19.2 |
|  | YBAB; hypothetical protein (1j8b_A) | 24.9 | 32 | 0.00089 | 19.1 |
| **Motif 9** | Protein NLRC5; CARD, RIG-I, protein binding (2mjm_A) | 41.3 | 28 | 0.00077 | 19.6 |
|  | Melittin; calcium-binding, EF-hand (3qrx_B) | 28.8 | 40 | 0.0011 | 14.5 |
|  | TENA family transcriptional regulator; TENA/THI-4/PQQC family (3mvu_A) | 27.7 | 68 | 0.0019 | 18.9 |
| **Motif 10** | CAMP-dependent protein kinase type II-alpha regulatory subunit (2izx_A) | 41.8 | 12 | 0.00034 | 18.4 |
|  | Receptor tyrosine-protein kinase ERBB-2; transmembrane helix dimer (2jwa_A) | 40.9 | 41 | 0.0012 | 16.9 |
|  | Major coat protein of PF1 virus (1pfi_A) | 39.5 | 49 | 0.0014 | 17.4 |
